# Reviewing the lines of therapy-concept in cancer treatment: a survey study among physicians

**DOI:** 10.1186/s12885-025-14940-0

**Published:** 2025-09-29

**Authors:** Lisa M. M. Falchetto, Bernd Bender, Ian Erhard, Stefanie Andreas, Linnea L. I. Schumann, Isabel Schnorr, Karin Berger-Thürmel, Jörg Janne Vehreschild, Daniel Maier

**Affiliations:** 1 Goethe University Frankfurt, Faculty of Medicine, Institute for Digital Medicine and Clinical Data Sciences, Theodor-Stern-Kai 7, Frankfurt am Main, 60590 Germany; 2 German Cancer Consortium (DKTK), Partner Site Frankfurt/Mainz, a partnership between DKFZ and University Medicine Frankfurt, Frankfurt Am Main, Germany; 3 Department I for Internal Medicine, University of Cologne, Faculty of Medicine and University Hospital Cologne, Cologne, Germany; 4 Department of Medicine III, University Hospital, Ludwig-Maximilians-University of Munich, Munich, Germany; 5 Bavarian Cancer Research Center (BZKF), Munich, Germany; 6 German Cancer Consortium (DKTK), Partner Site Munich a Partnership Between DKFZ and LMU University Hospital, Munich, Germany; 7 German Centre for Infection Research (DZIF), Partner Site Bonn-Cologne, Cologne, Germany

**Keywords:** Lines of therapy, Cancer treatment, Therapy planning, Survey study, Agreement

## Abstract

**Background:**

While the line of therapy (LOT) concept is crucial for clinical decision-making in oncology, the concept lacks a consensual understanding bearing the risk of miscommunication. This study examines physicians’ understanding of LOT, its perceived relevance, and the events and actions that determine a change of LOT within German oncological networks.

**Methods:**

Employing a snowball sampling approach, an anonymous online-administered survey was conducted among German physicians with oncological experience. Descriptive statistics and consensus metrics (Gini Mean Difference [GMD] and normalized entropy) were used to summarize the survey results and the relative agreement among the respondents, respectively.

**Results:**

Sixty physicians completed the survey. The majority were permanent members of an oncological tumor board (N = 46; 76.7%) and worked as a chief or senior physician (*N* = 37; 61.7%). Most respondents (*N* = 57, 95.0%) agreed that the LOT concept describes a planned therapeutic approach with a specified goal. Progression (*N* = 58; 96.7%), recurrence (N = 52; 86.7%), metastasis occurrence (*N* = 55; 91.7%), or severe side-effects (*N* = 46; 76.7%) were considered indicators prompting a change of LOT. Consideration of local therapeutic interventions (e.g., surgery (*N* = 20; 37.0%; GMD = 0.55, 95%-CI = [0.43; 0.64]) as independent LOT was widely disputed.

**Conclusions:**

This study reveals both commonalities and inconsistencies in physicians’ understanding of LOT in cancer treatment in Germany. Findings support the need for a greater standardization or refinement of the LOT concept which could help to minimize clinical misunderstandings and may enhance patient treatment strategies.

**Supplementary Information:**

The online version contains supplementary material available at 10.1186/s12885-025-14940-0.

## Background

Accurately determining a patient’s line of therapy (LOT) is crucial for planning subsequent anticancer treatment or assessing eligibility for clinical trials [1]. However, due to the lack of a consistently defined LOT concept accepted across disciplines, cancer types, and disease stages [2, 3], its interpretation in oncology practice remains variable, potentially resulting in communication challenges and misunderstandings [4, 5].

In clinical trials, lines of therapy (LOTs) have been defined for specific cancer entities such as multiple myeloma [1], advanced non-small cell lung cancer [2, 6], and metastatic colorectal cancer [7]. These definitions provide clear criteria for when a LOT should begin, end or be interrupted [1, 8] and whether local interventions (e.g., surgery, ablation, radiotherapy) are included [4, 9]. Such definitions are usually derived from treatment guidelines [1, 4, 6, 7], which define LOTs in a disease-specific and unambiguous manner. However, real-world practice often deviates from guideline recommendations, particularly in older or comorbid patients [2, 6, 10–12], and in late-line settings where evidence is limited [13–15]. As a result, guideline-based LOT definitions are not always sufficient for retrospective classification.

In medical data science, LOT is often treated as a retrospectively derivable algorithmic concept, e.g., for annotating LOT in electronic health records. Rules delineate LOT based on changes in anti-neoplastic medication and timing of administration, regardless of disease characteristics or treatment guidelines [16–18]. Patient cohorts are stratified by disease course and systemic anticancer treatment history [19–24]. Depending on research aims, such holistic approaches may risk clinical value.

Comparing these research strands reveals LOT’s contextual insufficiencies. An interview-based expert review by Falchetto et al. noted contested aspects, including uncertainties about systemic maintenance therapies and local interventions [5]. Experts struggled to assign maintenance therapies to a LOT when only part of a regimen is reused or when a different substance is introduced. Other studies criticized the exclusive systemic therapy focus in provisional LOT concepts, exposing a blind spot in standardizing LOT across therapy modalities [4, 21–23].

This survey study aims to determine how physicians in clinical oncology define a LOT and identify the specific therapeutic events they consider to mark a transition from one LOT to the next. It focuses on elements of the LOT concept previously noted as ambiguous, such as the classification of maintenance therapy, local interventions, or partial regimen changes, and analyzes how oncologists interpret and categorize these situations. The findings are intended to inform the development of a more consistent, comprehensive, and clinically applicable LOT definition.

## Methods

### Participants

The survey study targeted physicians practicing either in oncology or in another medical or surgical discipline with oncological patients. Respondents were recruited through scientific networks (i.e., German Cancer Consortium, [*German: Deutsches Konsortium für Translationale Krebsforschung* [DKTK]], and Oncological Research Data Center Project [*German: Onkologisches Forschungsdatenzentrum* [OnkoFDZ]]) and a medical society (i.e., German Society for Hematology and Medical Oncology [*German: Deutsche Gesellschaft für Hämatologie und Medizinische Onkologie* [DGHO]]). More specifically, individuals identified as occupying key roles within these networks and organizations were contacted and informed about the study. In line with snowball sampling methodology [25, 26], these individuals were asked to reach out to the target population, regardless of oncological focus or professional position, within their network and distribute the call for participation. Snowball sampling, which is usually employed to reach out to hard-to-access populations, was used to facilitate respondent recruitment. Assuming severe time limitations of the target population, our reasoning was that trusted leaders in scientific or clinical networks may strengthen the importance and prioritization of participation.

### Survey design

A questionnaire with a total of 50 questions was designed by multiple researchers with disciplinary backgrounds in medicine and social sciences. Questionnaire development was based on a qualitative study that involved a review of the relevant literature and a content analysis of interviews with expert senior physicians [5]. The relevant topics and questions were derived from this qualitative study, which were then transferred to the questionnaire in the form of closed questions and statements. The developers focused on clear operationalization and unambiguous perception of questions and answer scales. The questionnaire introduced the study, followed by a section asking about personal and professional background. Thereafter, survey questions assessed the agreement with statements concerning the definition and relevance of LOT (e.g.,”A line of therapy represents a largely self-contained therapy concept, chosen primarily based on various patient and tumor characteristics […]”, Response scale: “Fully agree”, “Rather agree”, “Undecided”, “Rather disagree”, “Strongly disagree”, “Cannot judge”) and events and actions initiating a change of LOT (e.g., “Progression of the primary tumor”).

Regarding the latter, fictitious patient scenarios were illustrated, involving maintenance therapy and different therapy modalities to assess their role in the change of LOT (e.g. “To what extent do the following therapeutic modalities […] represent a separate line of therapy? Surgery”, response scale: “Always”, “Mostly”, “Partially”, “Only in individual cases”, “Never”, “Cannot judge”, “I am not sure”). Finally, the survey explored the role of local therapies and therapy interruptions exemplified in various hypothetical scenarios. The complete questionnaire is provided in Additional file A.

Before launching the survey, the questionnaire was piloted by two physicians working in the field, including one trained oncologist, to test its practicability. The survey was conducted anonymously and was accessible via the online survey tool LimeSurvey (Version 3.28.9) from March to early June 2023. An access link to the questionnaire was distributed to the consortia and groups named above in March and a reminder was sent in April 2023 to recruit additional participants.

Ethics approval was obtained after review by the Ethics Committee of the Goethe University Frankfurt under reference number 274/18 and project number UCT-13–2022.

### Data processing and analysis

For data cleaning and preprocessing, the statistical programming language R (Version 4.2.2) and RStudio (Version 2023.12.1) were used.

Absolute and relative frequencies for all variables of the survey were calculated and reported. Missing values were excluded from calculations. Agreement among respondents was evaluated question-by-question, with separate analyses for binary, ordinal, and categorical (non-binary, non-ordinal) variables due to differing suitability of agreement measures.

For binary questions, affirmative response proportions, Clopper-Pearson (CP) 95%-confidence intervals (CIs) [27], and normalized entropy (NE) [28] were used to quantify uncertainty. A NE-value of $0$ corresponds to unanimity, while $1$ represents maximal diversity of opinions. In regard to the consensus metric NE must be pointed out, that this metric is very sensitive to a small number of dissenting opinions. For example, this could potentially lead to a high NE-value indicating dissent among respondents although the descriptive distribution shows a clear tendency.

The Gini mean difference (GMD) [29–31] was employed to assess agreement on ordinal questions. GMD and 95%-confidence interval (CI) were calculated after mapping the ordered categories to evenly spaced values on $\left[\text{0,1}\right]$. A GMD value of $0$ corresponds to unanimity, $2/3$ to maximal uncertainty (i.e., uniform distribution across answers), and $1$ to maximal dissension (i.e., equally sized groups opining at opposite ends of the spectrum). The GMD values were then standardized to allow comparison across questions with varying numbers of ordered answers.

For categorical questions, NE [28] estimates and Sison and Glaz' simultaneous 95%-CIs for multinomial proportions [32] were calculated.

A Benjamini-Hochberg (BH) correction [33] was applied across all question types. The false discovery rate (FDR) was managed at 5%, addressing multiple testing scenarios induced by testing the null hypothesis that distributions of expert opinions are uniformly diverse. An overview of all questions for calculating consensus metrics with the assigned detailed formulation is given in Table B.1 in Additional file B. Detailed procedures, interpretations, and data processing steps are elaborated in Tables B.2–B.5 in Additional file B, with mathematical definitions provided in Additional file C (Equations C.1–C.4).

## Results

### Respondents’ professional background and socio-demographics

As part of the data processing, two respondents were excluded because their answers indicated they were not part of the target population. Twenty-nine questionnaires were excluded since only socio-demographic information had been provided.

A total of 60 physicians either specialized in oncology or another medical or surgical discipline with a focus on oncological patients (e.g. neuro-oncology, thoracic oncology) participated in the survey. Most participants stated to be specialized in hematology/oncology (N = 42/60; 70.0%) and were permanent members of an oncological tumor board (N = 46/60; 76.7%).

The majority was between 36 and 55 years old (N = 42/60; 70.0%) and more than a third had 21 or more years of professional experience (N = 22/60; 36.7%). Additionally, more than half of the physicians worked as a chief or senior physician (N = 37/60; 61.7%) (Table 1).

**Table 1:** Personal and professional background of survey respondents

Personal and professional background	N (Percentages)
Age group	
25 or younger	0 (0.0%)
26–35	9 (15.0%)
36–45	21 (35.0%)
46–55	21 (35.0%)
56–65	8 (13.3%)
66 or older	1 (1.7%)
Professional experience	
None	0 (0.0%)
2 years or less	2 (3.3%)
3–5 years	7 (11.7%)
6–10 years	6 (10.0%)
11–15 years	13 (21.7%)
16–20 years	10 (16.7%)
21 years or more	22 (36.7%)
Professional position	
Assistant physician	9 (15.0%)
Specialized physician	14 (23.3%)
Chief/senior physician or other leading position	37 (61.7%)
Other job title	0 (0.0%)
Current department	
Department of Hematology/Oncology	42 (70.0%)
Oncological focus within another specialist area	9 (15.0%)
Department in which, among other things, oncological diseases are diagnosed/treated	9 (15.0%)
Other	0 (0.0%)
Experience with oncological tumor boards	
Hardly/None	0 (0.0%)
I present individual cases (10x/year or less)	4 (6.7%)
I regularly present cases (more than 10x/year)	10 (16.7%)
I am a permanent member	46 (76.7%)

### LOT: Definition and relevance

The vast majority of physicians (*N* = 57/60; 95.0%) agreed (fully or somewhat) that LOT is a self-contained therapy concept, based on various patient and tumor characteristics, including different therapy modalities, and aims at specific treatment goals. Although there was a relatively strong consensus regarding the definition of the LOT-concept (GMD = 0.28, 95%-CI = [0.21; 0.35]), regarding complex or rare situations, more than half of the respondents (*N* = 32/60; 53.3%) disagreed that there are clear-cut, uniform and commonly used criteria for determining LOTs (GMD = 0.52, 95%-CI = [0.42; 0.61]). While more than three quarters (*N* = 51/59; 86.5%) of physicians considered LOT as extremely relevant (defined as an answer of highly relevant or relevant) concept in everyday clinical practice (GMD = 0.41, 95%-CI = [0.32; 0.49]), half of respondents (*N* = 30/60; 50.0%; GMD = 0.38, 95%-CI = [0.31; 0.44]) deemed it as highly relevant for research purposes.

### Stage of disease and treatment intention

Most respondents considered LOT as a relevant concept across various disease stages, i.e. from early stages to locally advanced or metastatic disease (N = 41/60; 68.3%). However, opinions varied highly (GMD: 0.62, 95%-CI = [0.49; 0.76]).

A majority of respondents (N = 44/60; 73.3%) considered LOT relevant for both palliative and curative treatment intentions. Nevertheless, differing opinions on this question, led to a low level of agreement in responses (GMD: 0.54, 95%-CI = [0.40; 0.65]).

### Change of LOT

Most respondents stated that primary tumor progression (N = 58/60; 96.7%; NE: 0.21, 95%-CI = [0.04; 0.52]), metastasis occurrence (N = 55/60; 91.7%; NE: 0.41, 95%-CI = [0.18; 0.69]), disease recurrence (N = 52/60; 86.7%; NE: 0.57, 95%-CI = [0.32; 0.80]), and relevant therapy side-effects (N = 46/60; 76.7%; NE: 0.78, 95%-CI = [0.57; 0.94]) indicated changing LOTs.

Additionally, more than half of the respondents reported that discontinuation of all previously administered drugs (N = 37/54; 68.5%) equaled a change of LOT. A high NE-value of 0.90 (95%-CI = ([0.71; 0.99]), however, indicated strong disagreement. Furthermore, they predominantly stated that replacing previously administered drugs with different, non-equivalent drugs (N = 47/54; 87.0%; NE: 0.56, 95%-CI = [0.30; 0.81]) was a common practice for changing LOT. The majority of the surveyed physicians (N = 33/54; 61.1%) agreed that adding one or more new drugs to an existing drug regimen is an indicator of changing LOT. However, for this statement, analysis revealed a strong dissension (NE: 0.96, 95%-CI = [0.83; 1.00]) (Table 2). The frequencies, percentages and consensus metrics of all events and measures for a change of LOT are detailed in Table 2.

**Table 2:** Agreement on occurring events and actions initiating a change of lines of therapy

	Valid N	N (Percentages) of agreement	NE (95%-CI)
Which of the following events usually initiate a change of a line of therapy?			
Progression of the primary tumor	60	58/60 (96.7%)	0.21 [0.04, 0.52]
Metastasis of the primary tumor	60	55/60 (91.7%)	0.41 [0.18, 0.69]
Occurrence of a recurrence	60	52/60 (86.7%)	0.57 [0.32, 0.80]
Occurrence of relevant side-effects	60	46/60 (76.7%)	0.78 [0.57, 0.94]
Patient's preference	60	29/60 (48.3%)	1.00 [0.94, 1.00]
I cannot judge for any of the given answers	60	0/60 (0.0%)	0.00 [0.00, 0.33]
Which of the mentioned measures lead to a change of a line of therapy?			
Adding one or more new drugs to an existing drug regimen	54	33/54 (61.1%)	0.96 [0.83, 1.00]
Discontinuation of one or more drugs from an existing drug regimen	54	22/54 (40.7%)	0.98 [0.85, 1.00]
Discontinuation of all drugs administered so far	54	37/54 (68.5%)	0.90 [0.71, 0.99]
Replacement of a drug with another drug considered equivalent (from the same class of drugs)	54	9/54 (16.7%)	0.65 [0.40, 0.87]
Replacing the currently administered drugs with other drugs	54	47/54 (87.0%)	0.56 [0.30, 0.81]
Change in dose	54	9/54 (16.7%)	0.65 [0.40, 0.87]
Change in administration interval	54	8/54 (14.8%)	0.61 [0.35, 0.84]
Change in the route of administration	54	4/54 (7.4%)	0.38 [0.14, 0.68]
Interruption of treatment	54	12/54 (22.2%)	0.76 [0.53, 0.94]
I cannot judge this for any of the answers mentioned	60	6/60 (10.0%)	0.47 [0.23, 0.73]

Multiple answers were possible. Normalized entropy: $0$ corresponds to unanimity and $1$ represents maximal diversity of opinions

*Abbreviations*: *CI* Confidence interval, *NE* Normalized entropy

For all events and measures regarding the change of LOT, the proportions of affirmative responses and CP CIs are detailed and illustrated in Figure D.1 and Table D.1 in Additional file D.

**Fig. 1:**
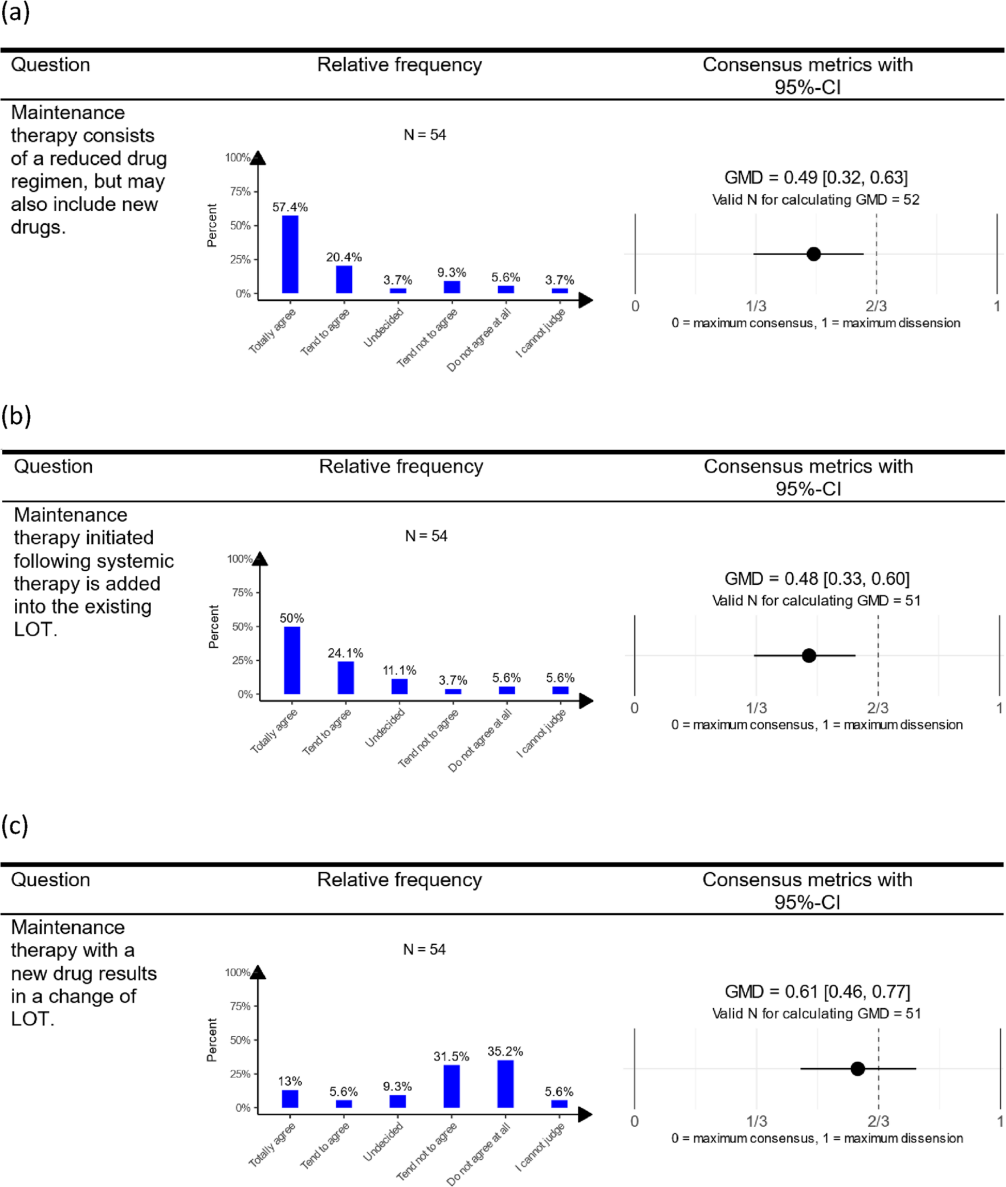
Descriptive and consensus results (95%-CIs) regarding maintenance therapy with reduced drug regimen or new drugs (**a**), maintenance therapy initiated after systemic therapy (**b**) and maintenance therapy with new drug as change of LOT (**c**). Note: A value for GMD of $0$ corresponds to unanimity, $2/3$ to maximal uncertainty (i.e., uniform distribution across answers), and $1$ to maximal dissension (i.e., equally sized groups opining at opposite ends of the spectrum). The standardization transformation serves to make GMD values comparable across questions with different numbers of possible answers. The response categories “I cannot judge” and “I am unsure” were excluded as indeterminate responses for calculating GMD. This leads to a little lower N for calculating the consensus metrics in comparison to the N for descriptive statistics. For the presentation in the table, a short version of the questions was used. An overview of the short versions with the assigned detailed formulation for all questions is given in Table B1 in Additional file B. Abbreviations: CI, confidence interval; GMD, Gini mean difference; LOT, line of therapy

### Maintenance therapy

A great part of the respondents (N = 42/54; 77.8%) agreed or tended to agree that maintenance therapy typically involves a reduced regimen of previously administered drugs but may also include new drugs. However, there was considerable heterogeneity across survey participants on this statement (GMD: 0.49, 95%-CI = [0.32; 0.63]). The majority (N = 40/54; 74.1%; GMD: 0.48, 95%-CI = [0.33; 0.60]) agreed that reduced regimen, initiated following systemic therapy, is part of an existing LOT. Conversely, two-third of physicians (N = 36/54; 66.7%; GMD: 0.61, 95%-CI = [0.46; 0.77]) did not consider the introduction of a new drug in systemic therapy as indicative of a LOT change. The results of all descriptive statistics and GMD values including CIs for all questions regarding maintenance therapy are visualized in Fig. 1.

### Therapy modality

**Fig. 2:**
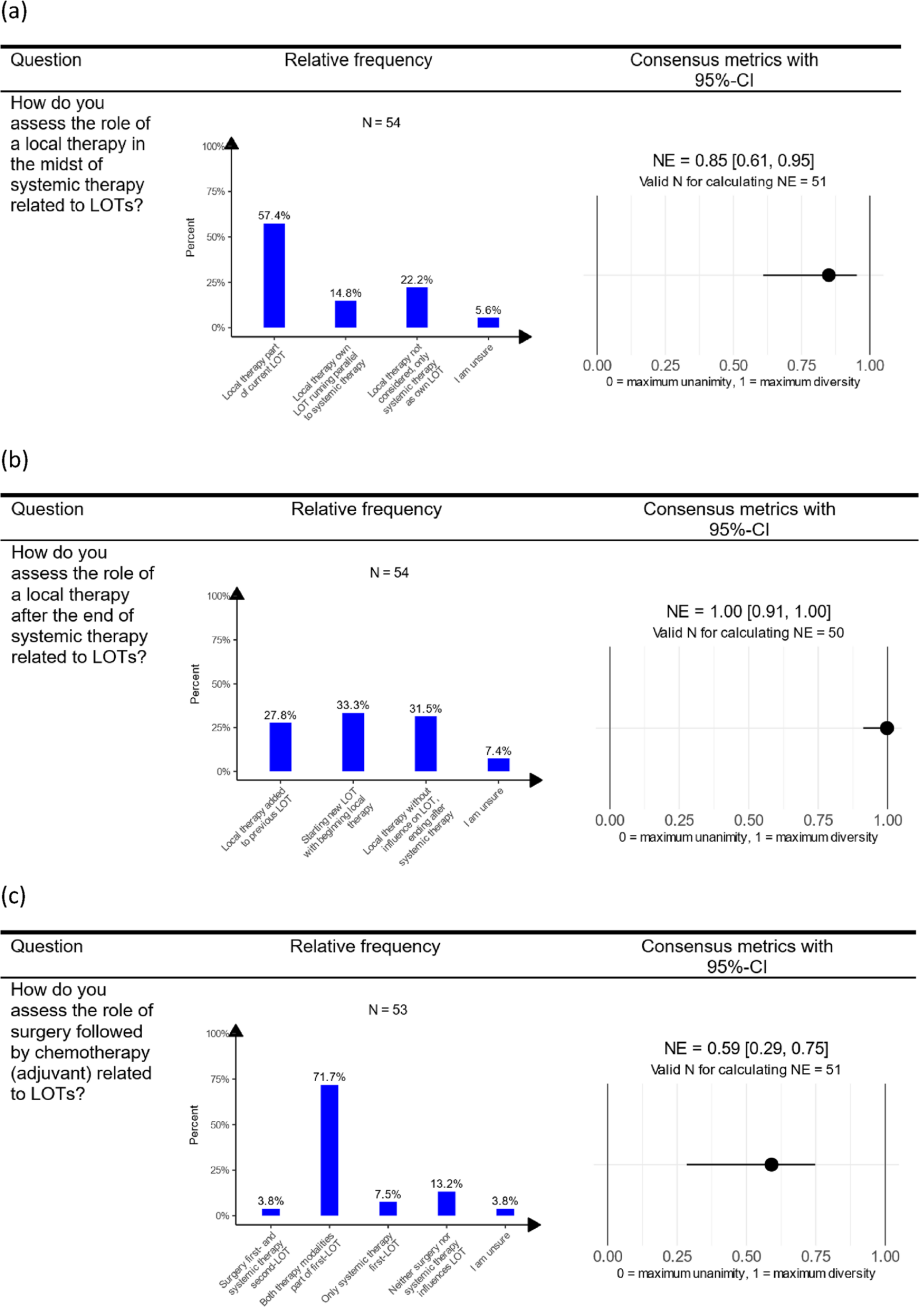
Descriptive and consensus results (95%-CIs) regarding local therapies in the midst of a systemic therapy (**a**), local therapies after the end of a systemic therapy (**b**) and surgery followed by chemotherapy (adjuvant) (**c**). Note: A value for NE of $0$ corresponds to unanimity, and $1$ represents maximal diversity of opinions. The confidence interval for the NE regarding local therapy after the end of systemic therapy now includes the value $1$, indicating that a hypothesis at the 5% significance level cannot be reject. This suggests that there is maximum disagreement about local therapy after the end of systemic therapy. The response category “I am unsure” was excluded as indeterminate response for calculating NE. This leads to a little lower N for calculating the consensus metrics in comparison to the N for descriptive statistics. For the presentation in the table, a short version of the questions was used. An overview of the short versions with the assigned detailed formulation for all questions is given in Table 1B in Additional file B. Abbreviations: CI, confidence interval; LOT, line of therapy; LOTs, lines of therapy; NE, normalized entropy

For most respondents, surgery (N = 20/54; 37.0%; GMD: 0.55, 95%-CI = [0.43; 0.64]) as well as radiotherapy (N = 23/54; 42.6%; GMD: 0.47, 95%-CI = [0.35; 0.57]) only partially constituted separate LOTs. A majority (N = 42/54; 77.8%; GMD: 0.36, 95%-CI = [0.17; 0.53]) approved that both radiotherapy and chemotherapy are essential to the LOT in cases of radiochemotherapy (Figure 2D in Additional file D).

Respondents further indicated that local therapy during systemic therapy is part of the ongoing LOT (N = 31/54; 57.4%; NE: 0.85, 95%-CI = [0.61; 0.95]). Opinions varied on local therapy post-systemic therapy: 27.8% (N = 15/54) considered local therapy as part of the previous LOT, 33.3% (N = 18/54) viewed it as the start of a new LOT, and 31.5% (N = 17/54) believed it does not impact the LOT status (NE: 1.00, 95%-CI = [0.91; 1.00]) (Fig. 2).

### Therapy interruption

In cases where therapy was interrupted for either 30 or 180 days across low- and high-grade tumors, most respondents indicated that the LOT remains unchanged post-interruption (30 days, low-grade: N = 50/53; 94.3%; GMD: 0.11, 95%-CI = [0.00; 0.24]; 180 days, low-grade: *N* = 30/53; 56.6%; GMD: 0.65, 95%-CI = [0.49; 0.92]; 30 days, high-grade: N = 44/53; 83.0%; GMD: 0.31, 95%-CI = [0.11; 0.49]; 180 days, high-grade: *N* = 24/52; 46.2%; GMD: 0.83, 95%-CI = [0.60; 0.99]).

However, if there was an interruption between two different therapies, either because of a therapy-free period in between (N = 46/52; 88.5%; GMD: 0.09, 95%-CI = [0.00; 0.19]) or due to progression or recurrence (*N* = 49/52; 94.2%; GMD: 0.14, 95%-CI = [0.00; 0.29]), most participants decided for a change of LOT (Table 3). All frequencies, percentages and consensus metrics for questions regarding the role of therapy interruptions for LOT are detailed in Table 3.

**Table 3:** Response behaviour regarding therapy interruptions in relation to lines of therapy based on hypothetical cases

Question	Response categories	N (%)	Valid N for calculating GMD	GMD (95%-CI)
30-day therapy interruption between the same therapy for low-grade tumors	The LOT remains the same after the interruption	50 (94.3%)	53	0.11 [0.00, 0.24]
	The LOT changes after the interruption	1 (1.9%)		
	I am not sure	2 (3.8%)		
	I cannot judge	0 (0.0%)		
	N total	53 (100.0%)		
180-day therapy interruption between the same therapy for low-grade tumors	The LOT remains the same after the interruption	30 (56.6%)	51	0.65 [0.49, 0.92]
	The LOT changes after the interruption	12 (22.6%)		
	I am not sure	9 (17.0%)		
	I cannot judge	2 (3.8%)		
	N total	53 (100.0%)		
30-day therapy interruption between the same therapy for high-grade tumors	The LOT remains the same after the interruption	44 (83.0%)	52	0.31 [0.11, 0.49]
	The LOT changes after the interruption	4 (7.6%)		
	I am not sure	4 (7.6%)		
	I cannot judge	1 (1.9%)		
	N total	53 (100.0%)		
180-day therapy interruption between the same therapy for high-grade tumors	The LOT remains the same after the interruption	24 (46.2%)	49	0.83 [0.60, 0.99]
	The LOT changes after the interruption	15 (28.9%)		
	I am not sure I cannot judge N total	10 (19.2%) 3 (5.8%) 52 (100.0%)		
Therapy interruption between two different therapies	The LOT remains the same after the interruption	0 (0.0%)	49	0.09 [0.00, 0.19]
	The LOT changes after the interruption	46 (88.5%)		
	I am not sure	3 (5.8%)		
	I cannot judge	3 (5.8%)		
	N total	52 (100.0%)		
Therapy interruption during therapy due to tumor progression/recurrence	The LOT remains the same after the interruption	20 (38.5%)	51	0.99 [0.66, 1.00]
	The LOT changes after the interruption	25 (48.1%)		
	I am not sure	6 (11.5%)		
	I cannot judge	1 (1.9%)		
	N total	52 (100.0%)		
Therapy interruption between two different therapies due to tumor progression/recurrence	The LOT remains the same after the interruption	2 (3.9%)	52	0.14 [0.00, 0.29]
	The LOT changes after the interruption	49 (94.2%)		
	I am not sure	1 (1.9%)		
	I cannot judge	0 (0.0%)		
	N total	52 (100.0%)		

The value $0$ of GMD std. corresponds to unanimity, $2/3$ to maximal uncertainty (ie, uniform distribution across answers), and $1$ to maximal dissension. To respect the ordered structure of the responses, the miscellaneous response “I cannot judge” was set aside to calculate GMD. Only category “I cannot judge” was excluded from calculating GMD, because “I am not sure” was interpreted as ordered response. The numbers of column “Valid N” relates only to the calculation of GMD

*Abbreviations*: *CI* Confidence interval, *GMD* Gini mean difference, *LOT* Line of therapy, *LOTs* Lines of therapy

All non-standardized GMD-values for the various thematic blocks are listed in Table D.2 in Additional file D.

## Discussion

To our knowledge, this is the first survey study with oncological focus to assess physicians’ understandings of the “line of therapy”-concept regarding its defining criteria for change of LOT. Therefore, we have drawn on the expertise of a larger group of physicians with oncological expertise in Germany. Responses are based on many years of clinical experience and therefore provide significant insights on the topic.

Our study underscores that the majority of physicians consider LOT as a relevant concept for their oncological practice. Most respondents confirmed a current lack of universal applicable definitions, which was mirrored by heterogenous assessment of specific situations assessed in our study. Our results highlight significant and concrete uncertainties concerning specific criteria and parameters relevant for the definition and operationalization of the LOT concept. Ambiguous response patterns prevailed, for example, concerning the relevance of various therapy modalities within a patient’s course of treatment.

The surveyed example cases have put forth the ambiguous nature of the LOT concept with respect to, e.g., the role of local therapies or treatment interruptions. For instance, while a local therapy during a systemic therapy was considered as part of an ongoing LOT, a local therapy after a systemic therapy was rated by over a third of respondents as start of a new LOT. The ambiguity of treatment interruptions was indicated by the influence of progression or recurrence during the interruption on LOT, whereas duration alone did not affect LOT. Based on our findings, future research should aim to explicitly operationalize these criteria related to specific therapeutic constellations and disease entities in question.

In summary, our results show that LOT are considered relevant in German oncological networks with respect to palliative and curative treatment intentions, which is in line with findings from an earlier study [4]. Progression, recurrence, the occurrence of metastases and significant side effects were identified as key indicators signaling a change of LOT. These indicators for the transition from one LOT to another were also congruent with qualitative research findings [5] and existing algorithmic applications [8, 19]. Furthermore, our results confirm that the addition of a new drug is seen as criterion for a change of LOT, whereas the substitution of previously administered drugs with equivalent ones is not considered to lead to a LOT change [34].

Local therapies were only partially considered as separate LOTs, which does not imply local therapies to be irrelevant for LOT definitions. Their exact role in LOT remains unclear, aligning with prior research findings [4]. Similarly, the impact of therapy interruptions on LOT is ambiguous, which is proven by questions with both strong consensus and strong dissension among the physicians. Thus, their implications are specific to individual scenarios, reflecting variability noted in earlier studies [6, 8, 23, 24, 35]. This vagueness reveals the necessity to assess and clarify the roles of local therapies and therapy interruptions in the future. Local therapies could also be important as components of LOT besides systemic therapies, whereas therapy interruptions have a strong impact on the classification of LOT.

There are limitations that need to be considered regarding the generalizability of our results. First, despite the great effort that went into distributing the questionnaire and reminding the networks to ask for participation repeatedly, the sample size (N = 60) remained relatively small. While a larger sample would imply greater confidence in the results, we deem the sample size sufficiently large for the intended purpose of exploration. Second, the applied snowball sampling method is prone to result in a selection bias which likely impairs the representativity of the results. We deem a sampling bias as very likely with respect to the professional backgrounds of the respondents: the applied snowball-sampling technique led to a respondent sample that overrepresents senior consultants and physicians in leadership positions with extensive professional experience and regular participation in oncological tumor boards. Due to this likely selection bias, we decided against conducting in-depth comparative analyses between demographic and/or professional subgroups which would likely have led to invalid conclusions. Therefore, we hypothesize that the heterogeneity of responses would have been considerably higher if more less experienced physicians would have been participating. In addition, it is possible that language concepts may be interpreted differently in other countries and health systems; since the survey was only spread through German communication channels, transferability to other countries and health systems needs to be confirmed in future studies. To improve generalizability, future studies should try to employ random sampling on a predefined population of physicians and if possible, aim for a comparison across countries. Furthermore, although the questionnaire has been pre-tested, no further validation of the applied scales has been pursued which presents a potential limitation of the applied method.

Another possible limitation of the survey study is that participants were asked to rate generic therapeutic concepts, not specific examples. This may have limited their ability to transfer the questions to real-world examples from their respective practice and thus influenced towards stronger abstraction of the answers. However, since we were specifically trying to understand governing principles instead of the assessment of a specific therapeutic situation, we maintain our approach as valid for our objective.

In oncology practice, determining LOT is essential for selecting patients’ treatment options, deciding about clinical trial eligibility and quality audit documentation. While treatment guidelines may provide orientation for determining LOT, real-world studies have shown that patient pathways often deviate from these prescribed patterns [6, 10]. Without such orientation, clinical heuristics based on experiences and implicit rules prone to misunderstandings are likely used to guide action when LOT need to be determined.

## Conclusions

In conclusion, this study significantly advances the understanding of the complexities in defining and classifying LOTs in cancer treatment and raises the question whether LOT are a reliable means of communicating patient status between physicians. By identifying knowledge gaps and inconsistencies in current definitions and criteria, our results highlight the need for standardizing concepts of LOT. Given that future research corroborates our findings, areas of the agreement (“the common ground”) about LOT can be used as an initial point for a further clarification and disambiguation of the concept. A systematic process (e.g., Delphi-method) could be applied to approximate consensus among experts that, if carried further, might initialize a broader discussion in the scientific and clinical communities. The presumed clinical relevance of LOT lies first and foremost in its predictive value for patient outcomes. Therefore, a re-assessment of the LOT concept and its variants, based on large, socio-demographically and professionally balanced samples, is required to evaluate this predictive potential and should be expanded internationally to compare our findings from Germany with those from other countries. Such a re-assessment should evaluate LOT in comparison to alternative measures of a patient’s therapy status. Such advancements are necessary for improving communication between different care providers, minimizing clinical misunderstandings, optimizing patient treatment strategies, standardizing care procedures across oncology and ultimately improving patient outcomes.

## Supplementary Information

Supplementary Material 1.

## Data Availability

An anonymized version of the dataset used in this study is available from the corresponding author (Bernd Bender, E-Mail: bernd.bender@dkfz-heidelberg.de) upon reasonable request.

## Abbreviations

LOT: Line of therapy
LOTs: Lines of therapy
DKTK: German Cancer Consortium
OnkoFDZ: Oncological Research Data Center
DGHO: German Society for Hematology and Medical Oncology
CP: Clopper-Pearson
Cis: Confidence intervals
NE: Normalized entropy
GMD: Gini mean difference
CI: Confidence interval
BH: Benjamini-Hochberg
FDR: False discovery rate
