# Supplementary material for: Reviewing the lines of therapy-concept in cancer treatment: a survey study among physicians
Source: BMC Cancer. 2025 Sep 29;25:1439. doi: 10.1186/s12885-025-14940-0 (PMC12482237; doi:10.1186/s12885-025-14940-0)
Supplement: Supplementary file 1 — Supplementary Material 1. [file 12885_2025_14940_MOESM1_ESM.docx]

**Additional files**

**Additional file A**

**Table A.1.** Questionnaire Lime Survey.

Line of therapy concepts in oncology

Description:

This survey is part of a doctoral thesis at Goethe University Frankfurt am Main. It targets medical professionals involved in the care of oncology patients. The survey aims to provide insight into the various therapeutic approaches for treating solid and non-solid cancers and to establish a basis for decision-making in defining criteria for lines-of-therapy approaches.

Welcome Message:

Dear survey participants,

Thank you for your interest and time! Your participation contributes greatly to our project.

In this survey, you will be asked questions on various aspects related to "Therapeutic Approaches in Oncology." There are no right or wrong answers; we aim to capture your personal views. To ensure the study's success, please complete the questionnaire fully without skipping any questions. All data collected will remain anonymous and treated with strict confidentiality.

The project aims to explore different understandings of therapeutic approaches among oncology professionals. Your input will help create a basis for defining criteria for these approaches.

We appreciate your participation!

Lisa Falchetto, Daniel Maier, and Janne Vehreschild

There are 26 questions in this survey.

Closing Message:

Thank you for taking part in the survey!

Your responses have been saved.

Data Protection Rights

How is your data handled and protected?

Your data will be used solely for the purpose described above. Responses are anonymized, analyzed, and securely stored at the German Cancer Research Center (DKFZ) in Germany (Heidelberg). When the results are published, no reference to your identity can be made.

What are your data protection rights?

The legal basis for data processing is your consent, as outlined in Article 6, paragraph 1, letter a, and Article 9, paragraph 2, letter a of the General Data Protection Regulation. The DKFZ is the data controller as per the General Data Protection Regulation. You have the right to withdraw your consent at any time without providing a reason by selecting "Leave survey and delete responses" (located at the top right of the header on each page of the questionnaire). In such a case, all information provided up to that point will be deleted. Once the questionnaire is submitted in full, it is not possible to withdraw consent since individual questionnaires and data cannot be personally identified. Therefore, you will no longer be able to request information about data stored by the DKFZ, or request deletion, correction, or restriction of processing.

If you have concerns regarding data processing or compliance with data protection, you can contact the Data Protection Officer at the German Cancer Research Center:

Data protection supervisor

German Cancer Research Center

Im Neuenheimer Feld 280

69120 Heidelberg

Phone: 06221 / 420

E-mail: [datenschutz@dkfz.de](mailto:datenschutz@dkfz.de)

You also have the right to lodge a complaint with any data protection supervisory authority. A list of supervisory authorities in Germany can be found at <https://www.bfdi.bund.de/DE/Infothek/Anschriften_Links/anschriften_links-node.html>

Participation

Your participation in this survey is voluntary. By reading and understanding this information along with the declaration of consent, you confirm your willingness to participate by accepting our privacy policy through ticking the box provided. Only then can you proceed to answer the survey questions. Your consent becomes effective upon submission of the form.

Should you have any further inquiries about the project or any other aspects of the survey, please feel free to reach out to the following contacts:

Phone: 069 / 6301 86596

E-Mail: daniel.maier@dkfz.de (Subject: "Line of Therapy Concepts in Oncology")

Dr. Daniel Maier is responsible for the survey.

E-Mail: daniel.maier@dkfz.de

We appreciate your interest and support.

Yours sincerely,

Daniel Maier

Please choose one of the following answers:

Please select only one of the following answers:

⃝ I have read the information and accept the privacy policy.

Personal and professional background

In the following, we will ask you about your personal and professional background as well as your level of experience in the care of oncological patients.

Which of the following age categories (in years) do you belong to?

Please choose one of the following answers:

⃝ 25 or younger

⃝ 26 – 35

⃝ 36 – 45

⃝ 46 – 55

⃝ 56 – 65

⃝ 66 or older

How many years of professional experience do you have in the care of oncology patients?

Please choose one of the following answers:

⃝ None

⃝ 2 or less

⃝ 3 – 5

⃝ 6 – 10

⃝ 11 – 15

⃝ 16 – 20

⃝ 21 or more

Which professional position applies to you?

Please choose one of the following answers:

⃝ Assistant physician

⃝ Specialized physician

⃝ Chief / senior physician or other leading position

⃝ Other job title (please specify in comment box)

Please leave a comment on your selection

In which department are you currently working?

Please choose one of the following answers:

⃝ Department of Hematology/Oncology

⃝ Oncological focus within another specialist area (please specify in the comment field)

⃝ Department in which, among other things, oncological diseases are diagnosed/treated (please specify in the comment field)

⃝ Other (please specify in the comment field)

Please leave a comment on your selection

How much experience do you have with oncological tumor boards?

Please choose one of the following answers:

⃝ Hardly/None

⃝ I present individual cases (10x per year or less) at a tumor board.

⃝ I regularly present cases (more than 10x per year) at a tumor board.

⃝ I am a permanent member of a tumor board.

Line-of-therapy-concept

In the following, we would like to understand your interpretation of the concept of line of therapy. Specifically, we are interested in what constitutes a line of therapy for you and how relevant you consider this concept overall.

To what extent do you agree with the following definition of the line-of-therapy-concept?

A line of therapy represents a largely self-contained therapy concept, chosen primarily based on various patient and tumor characteristics. It can include different therapy modalities and specific treatment goals, such as tumor control or cancer cure.

Please choose one of the following answers:

⃝ Fully agree

⃝ Rather agree

⃝ Undecided

⃝ Rather disagree

⃝ Strongly disagree

⃝ Cannot judge

Please leave a comment on your selection

To what extent do you agree with the following statement?

There are currently clear and uniform criteria in place to determine the lines of therapy even in rare and complex situations in the course of the patient.

Please choose one of the following answers:

⃝ Fully agree

⃝ Rather agree

⃝ Undecided

⃝ Rather disagree

⃝ Strongly disagree

⃝ Cannot judge

We are interested in your assessment.

Please select the appropriate answer for each item:

|  | Highly relevant | Relevant | Partially relevant | Not very relevant | Not relevant at all |
| --- | --- | --- | --- | --- | --- |
| How relevant do you consider the definition of lines of therapy in everyday clinical practice? | 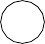 | 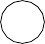 | 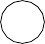 | 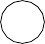 | 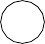 |
| How relevant do you think it is to define the lines of therapy in research, e.g. in the context of studies? | 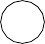 | 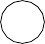 | 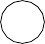 | 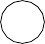 | 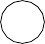 |

To what extent do you agree with the following statements?

Please select the appropriate answer for each item:

|  | Fully agree | Rather agree | Undecided | Rather disagree | Strongly disagree | Cannot judge |
| --- | --- | --- | --- | --- | --- | --- |
| Lines of therapy are only relevant in inoperable, locally advanced or metastatic cancers, but not in early stages of the disease. | 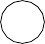 | 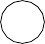 | 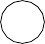 | 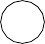 | 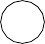 | 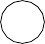 |
| Lines of therapy are only relevant in the context of a palliative treatment, but not in the context of a curative therapy. | 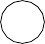 | 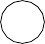 | 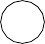 | 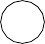 | 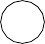 | 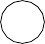 |

Change in line of therapy

This block of questions deals with the ending of one line of therapy and the beginning of a new one.

Which of the following events usually initiate a change of a line of therapy?

Please select the answers that apply:

🞎 Progression of the primary tumor

🞎 Metastasis of the primary tumor

🞎 Occurrence of a recurrence

🞎 Occurrence of relevant side-effects

🞎 Patient's preference

🞎 I cannot judge for any of the given answers.

🞎 Other:

Which of the mentioned measures, if applicable as a consequence of the events you selected in question 10, lead to a change in lines of therapy?

*"Drugs" include chemotherapy, immunotherapy, and hormone therapy.*

Please select the answers that apply:

🞎 Adding one or more new drugs to an existing drug regimen

🞎 Discontinuation of one or more drugs from an existing drug regimen

🞎 Discontinuation of all drugs administered so far

🞎 Replacement of a drug with another drug considered equivalent (from the same class of drugs)

🞎 Replacing the currently administered drugs with other drugs

🞎 Change in dose

🞎 Change in administration interval

🞎 Change in the route of administration

🞎 Interruption of treatment

🞎 I cannot judge this for any of the answers mentioned.

🞎 Other:

Maintenance therapy

In the following, we ask for your assessment of the role of maintenance therapy in relation to lines of therapy.

To what extent do you agree with the following statements?

Please select the appropriate answer for each item:

|  | Totally agree | Rather agree | Undecided | Rather disagree | Strongly disagree | Cannot judge |
| --- | --- | --- | --- | --- | --- | --- |
| Maintenance therapy usually consists of a reduced drug regimen of the drugs previously administered, but may also include new drugs. | 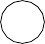 | 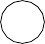 | 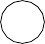 | 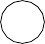 | 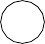 | 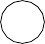 |
| If maintenance therapy, which typically involves a reduced drug regimen, is initiated following systemic therapy, it is added into the existing line of therapy. | 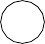 | 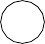 | 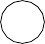 | 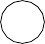 | 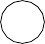 | 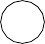 |
| If maintenance therapy involves a new drug that has not yet been used in the previous systemic therapy, this results in a change of the line of therapy. | 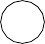 | 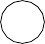 | 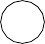 | 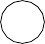 | 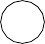 | 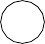 |

Therapy modalities

In the following section, we ask for your evaluation of various therapy modalities concerning their role in different lines of therapy, based on several case studies.

To what extent do the following therapeutic modalities (possibly in connection with other therapy modalities) represent a separate line of therapy?

Please select the appropriate answer for each item:

|  | Always | Mostly | Partially | Only in  individual cases | Never | Cannot judge | I am not sure |
| --- | --- | --- | --- | --- | --- | --- | --- |
| Surgery | 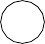 | 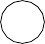 | 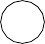 | 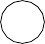 | 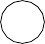 | 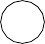 | 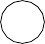 |
| Radiotherapy | 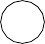 | 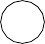 | 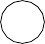 | 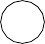 | 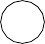 | 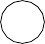 | 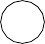 |
| Regional treatment procedures (e.g. TACE) | 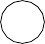 | 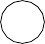 | 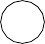 | 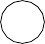 | 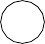 | 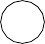 | 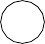 |

To what extent do you agree with the following statement?

In the case of simultaneous radiochemotherapy, both radiotherapy and chemotherapy are part of the line of therapy.

Please choose one of the following answers:

⃝ Totally agree

⃝ Rather agree

⃝ Undecided

⃝ Rather disagree

⃝ Strongly disagree

⃝ Cannot judge

Based on the following hypothetical case, how do you assess the role of local therapies related to lines of therapy?


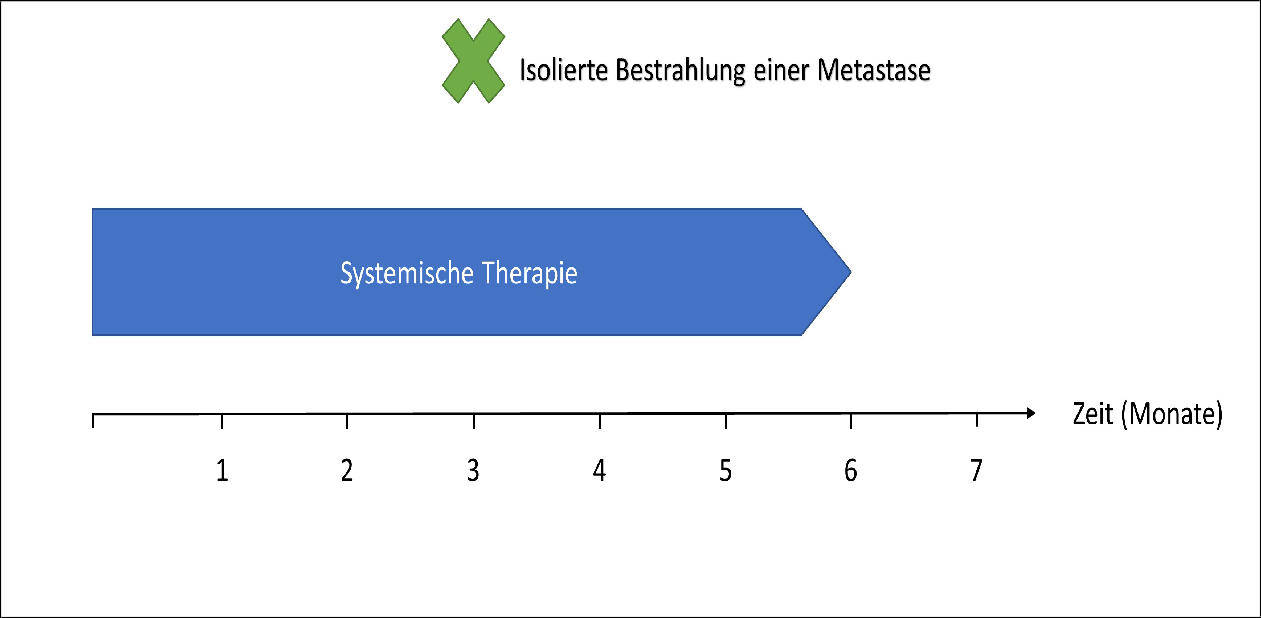


Isolated irradiation of a metastasis

Time (months)

Systemic therapy

Please choose one of the following answers:

⃝ Local therapy is part of the ongoing line of therapy.

⃝ Local therapy is a separate line of therapy that runs parallel to systemic therapy.

⃝ Local therapy is not taken into account. Only systemic therapy is a line of therapy in its own right.

⃝ I am not sure.

⃝ Cannot judge.


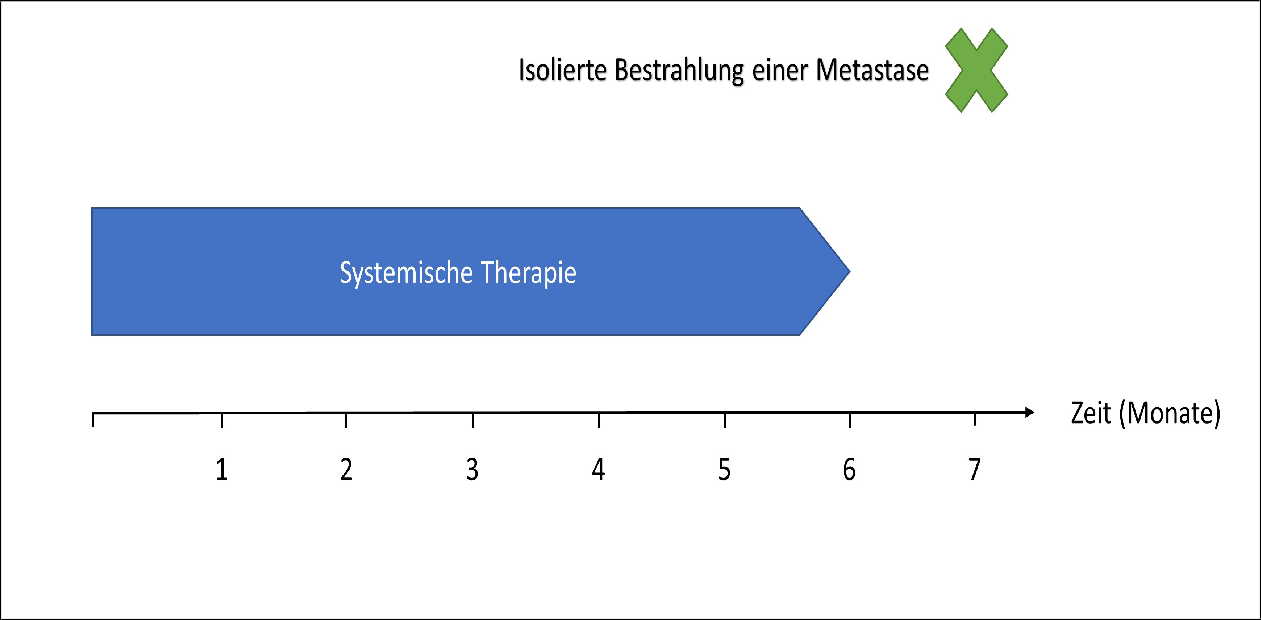
Based on the following hypothetical case, how do you assess the role of local therapies related to lines of therapy?

Isolated irradiation of a metastasis

Systemic therapy

Time (months)

Please choose one of the following answers:

⃝ Local therapy is added to the previous line of therapy.

⃝ Initiation of local therapy marks the beginning of a new line of therapy.

⃝ After completing systemic therapy, the line of therapy concludes, and local therapy does not have any further impact.

⃝ I am not sure.

⃝ Cannot judge.

Based on the following hypothetical case, how do you assess the role of local therapies related to lines of therapy?


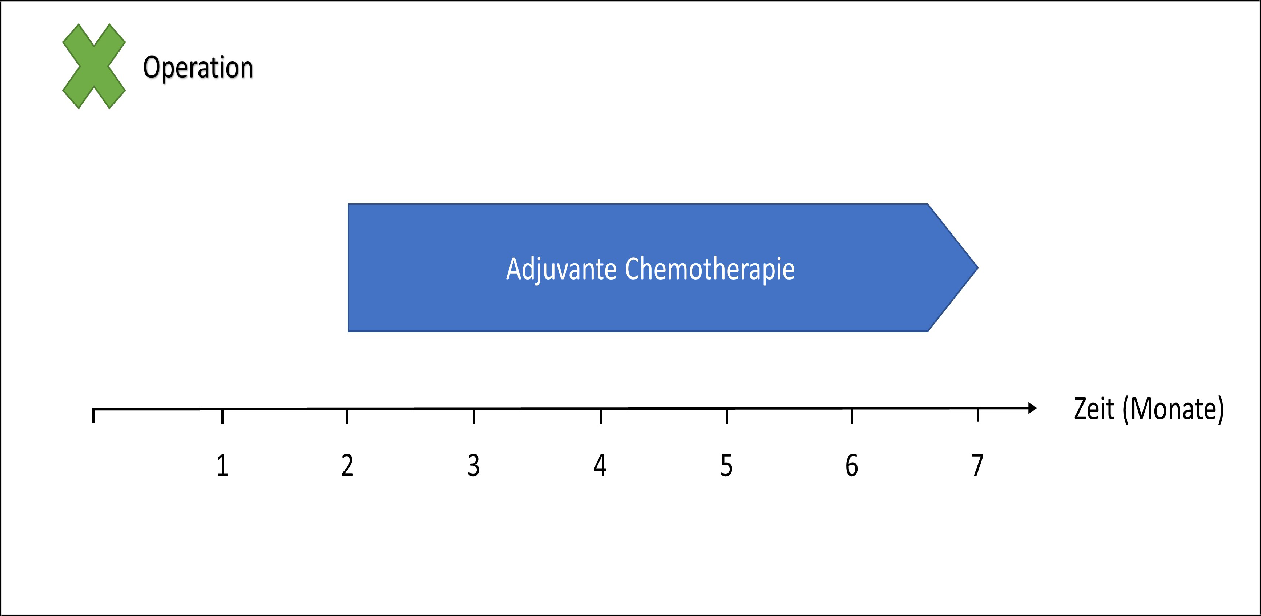


Surgery

Adjuvant chemotherapy

Time (months)

Please choose one of the following answers:

⃝ Surgery is the first-line therapy, and the systemic therapy is the second-line therapy.

⃝ Both therapy modalities are part of first-line therapy.

⃝ Only systemic therapy is considered first-line therapy.

⃝ Neither surgery nor systemic therapy has any influence on the lines of therapy.

⃝ I am not sure.

⃝ Cannot judge.

Therapy interruption

In the following, we ask for your assessment of the role of therapy interruptions in relation to lines of therapy on the basis of several case studies.

***CAVE: These are not scheduled interruptions between two cycles.***

Based on the following hypothetical case, how do you assess the role of therapy interruptions in relation to lines of therapy?


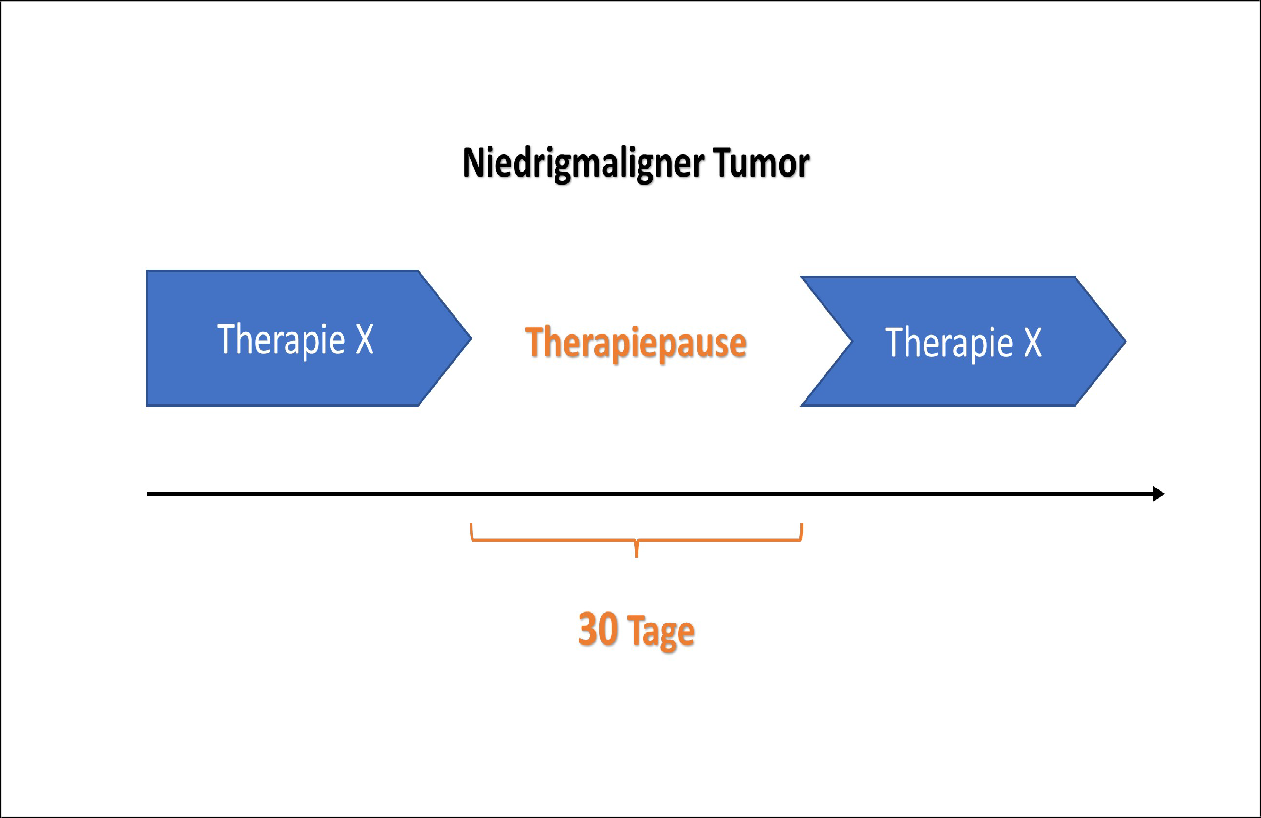


**Low-grade tumor**

Therapy interruption

Therapy X

Therapy X

**30 days**

Please choose one of the following answers:

⃝ The line of therapy remains the same after the interruption.

⃝ The line of therapy changes after the interruption.

⃝ I am not sure.

⃝ Cannot judge.

Based on the following hypothetical case, how do you assess the role of therapy interruptions in relation to lines of therapy?


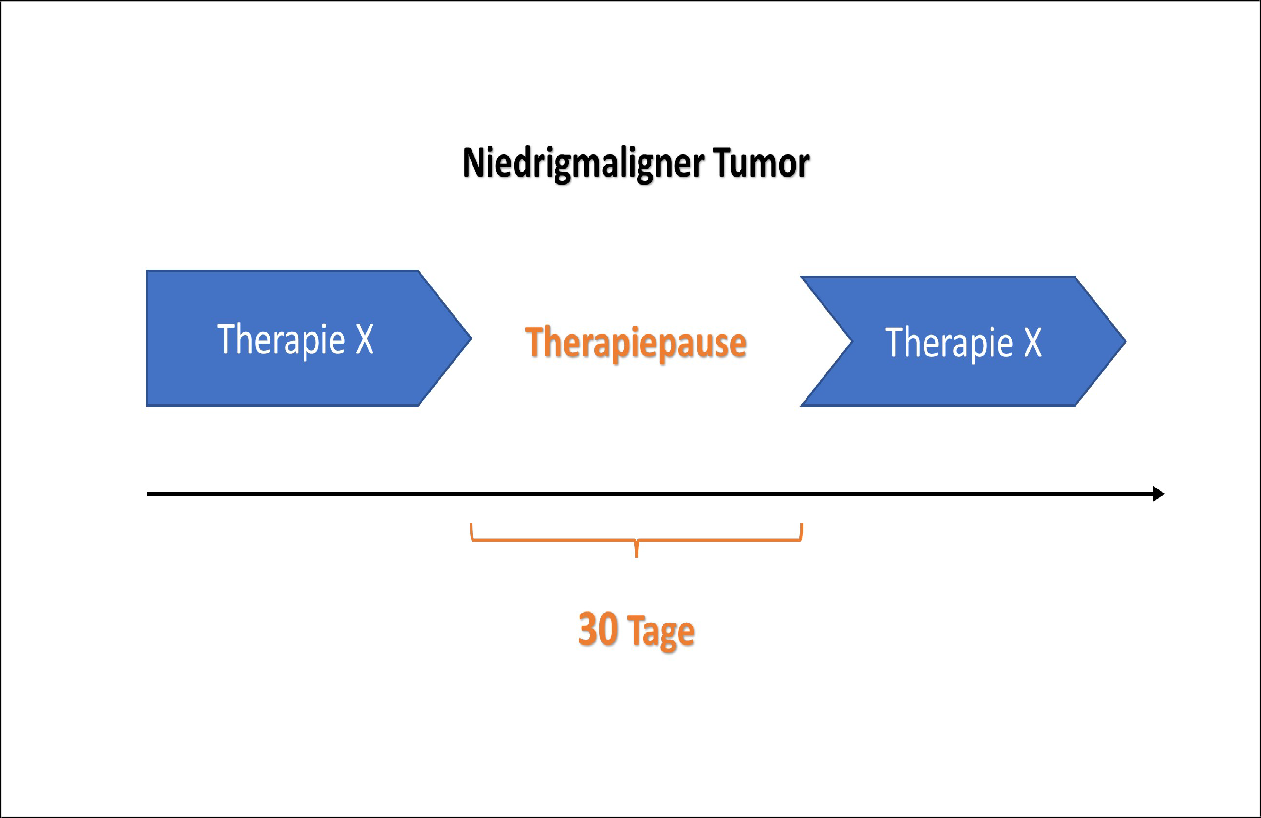


**Low-grade tumor**

Therapy interruption

Therapy X

Therapy X

**180 days**

Please choose one of the following answers:

⃝ The line of therapy remains the same after the interruption.

⃝ The line of therapy changes after the interruption.

⃝ I am not sure.

⃝ Cannot judge.

Based on the following hypothetical case, how do you assess the role of therapy interruptions in relation to lines of therapy?


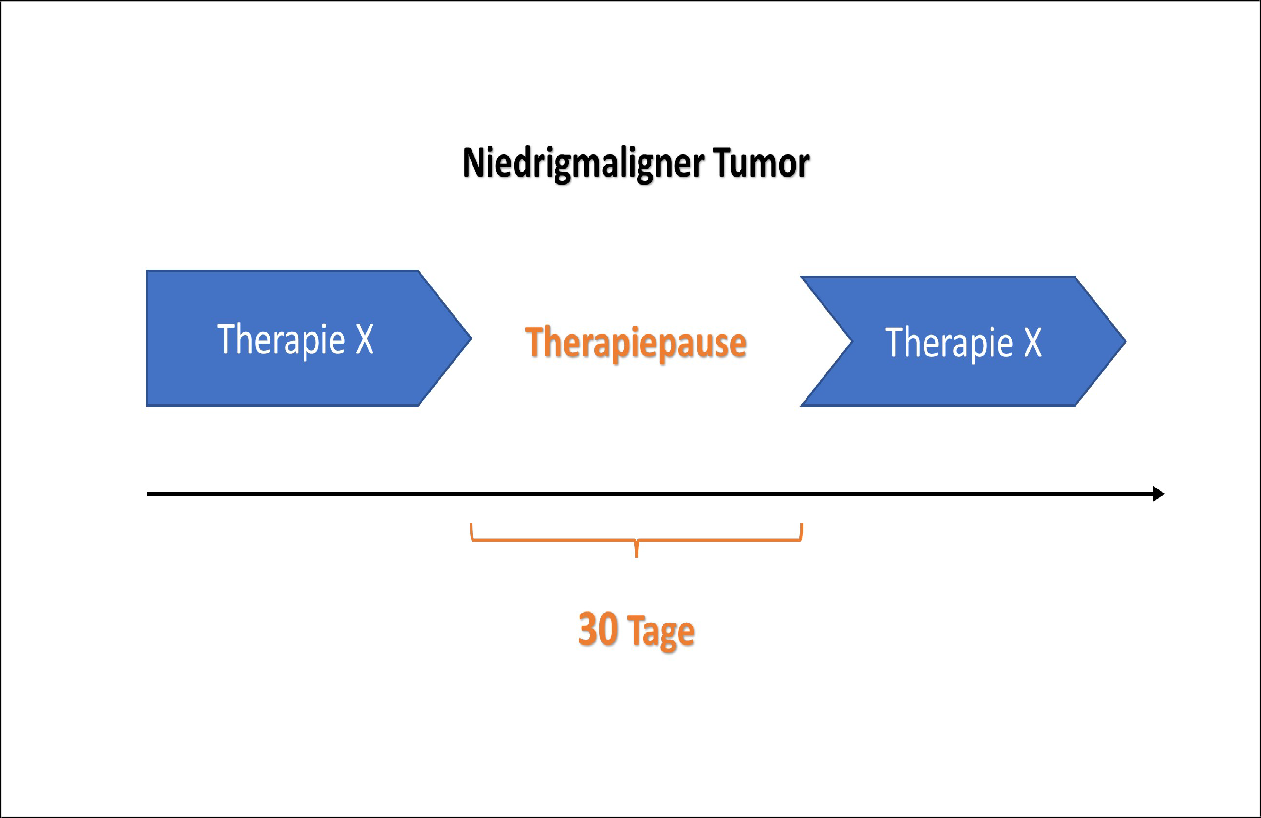


**High-grade tumor**

Therapy interruption

Therapy X

Therapy X

**30 days**

Please choose one of the following answers:

⃝ The line of therapy remains the same after the interruption.

⃝ The line of therapy changes after the interruption.

⃝ I am not sure.

⃝ Cannot judge.

Based on the following hypothetical case, how do you assess the role of therapy interruptions in relation to lines of therapy?


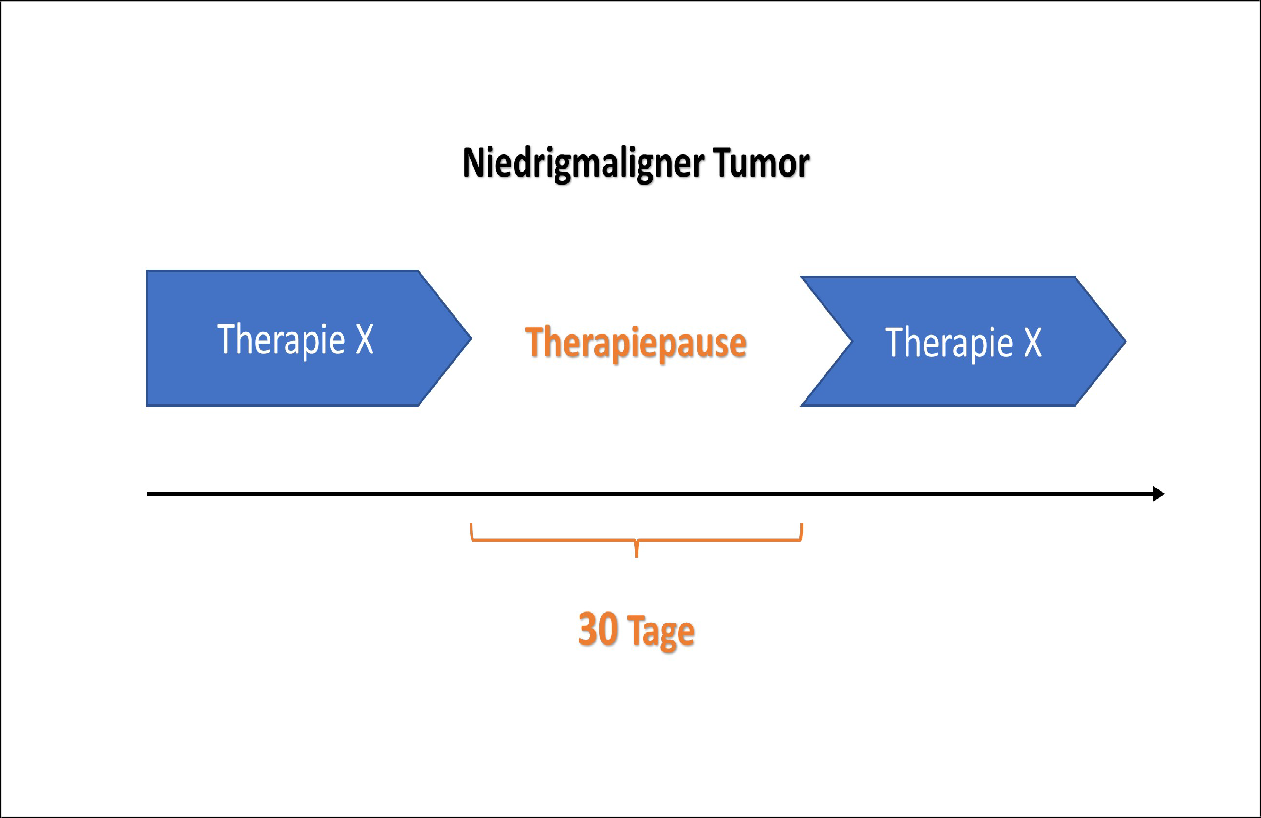


**High-grade tumor**

Therapy interruption

Therapy X

Therapy X

**180 days**

Please choose one of the following answers:

⃝ The line of therapy remains the same after the interruption.

⃝ The line of therapy changes after the interruption.

⃝ I am not sure.

⃝ Cannot judge.

Based on the following hypothetical case, how do you assess the role of therapy interruptions in relation to lines of therapy?


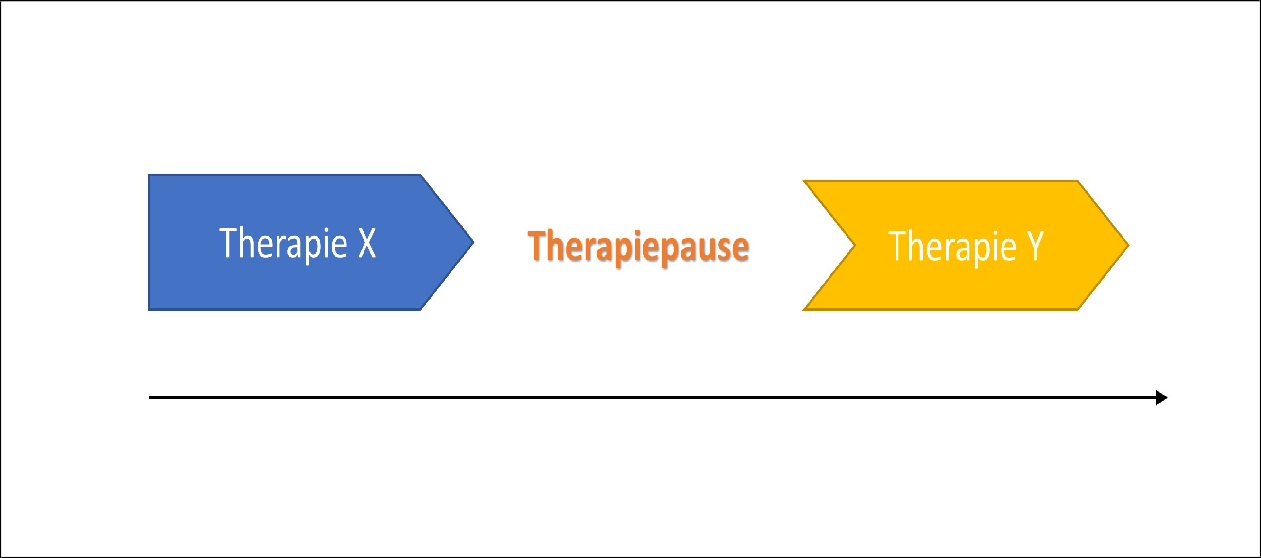


Therapy interruption

Therapy Y

Therapy X

Please choose one of the following answers:

⃝ The line of therapy remains the same after the interruption.

⃝ The line of therapy changes after the interruption.

⃝ I am not sure.

⃝ Cannot judge.

Based on the following hypothetical case, how do you assess the role of therapy interruptions in relation to lines of therapy?


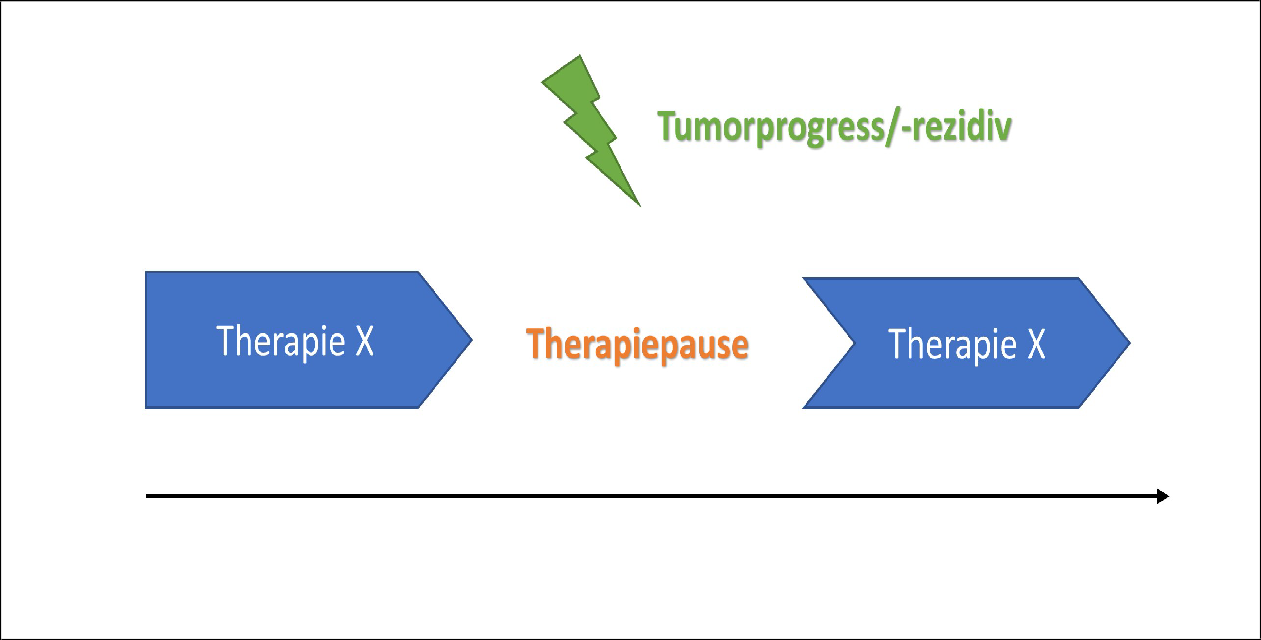


**Tumor progression/recurrence**

Therapy interruption

Therapy X

Therapy X

Please choose one of the following answers:

⃝ The line of therapy remains the same after the interruption.

⃝ The line of therapy changes after the interruption.

⃝ I am not sure.

⃝ Cannot judge.

Based on the following hypothetical case, how do you assess the role of therapy interruptions in relation to lines of therapy?


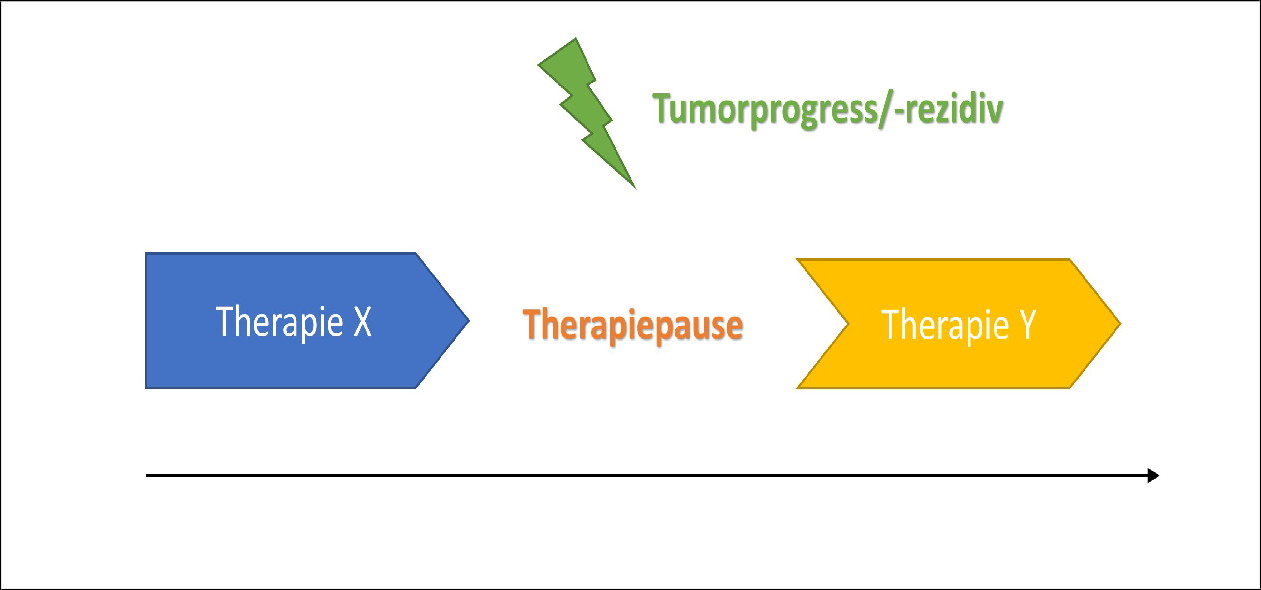


**Tumor progression/recurrence**

Therapy interruption

Therapy Y

Therapy X

Please choose one of the following answers:

⃝ The line of therapy remains the same after the interruption.

⃝ The line of therapy changes after the interruption.

⃝ I am not sure.

⃝ Cannot judge.

What is the duration of a therapy interruption (in weeks) after which it is definitely considered the end of therapy, leading to the assumption of a change in the line of therapy when treatment resumes?

Please enter your answer here:

Thank you for your participation in the survey!

Your answers have been saved.

Submission of your completed questionnaire:

Thank you for answering the questionnaire.

**Additional file B**

**Table B.1.** Overview of questions for calculating consensus metrics with short and detailed version.

| **Short version for question** | **Detailed version for question** |
| --- | --- |
| 1. LOT is a self-contained concept. | To what extent do you agree with the following definition of the line-of-therapy-concept? A line of therapy represents a largely self-contained therapy concept, chosen primarily based on various patient and tumor characteristics. It can include different therapy modalities and specific treatment goals, such as tumor control or cancer cure. |
| 1. LOT-criteria are given in rare / complex situations. | To what extent do you agree with the following statement? There are currently clear and uniform criteria in place to determine the lines of therapy even in rare and complex situations in the course of the patient. |
| 1. How relevant is the definition of LOTs in everyday clinical practice? | How relevant do you consider the definition of lines of therapy in everyday clinical practice? |
| 1. How relevant is it to define LOTs in research? | How relevant do you think it is to define the lines of therapy in research, e.g. in the context of studies? |
| 1. LOTs are only relevant in inoperable, locally advanced / metastatic cancers. | To what extent do you agree with the following statements? Lines of therapy are only relevant in inoperable, locally advanced or metastatic cancers, but not in early stages of the disease. |
| 1. LOTs are only relevant for palliative treatment. | To what extent do you agree with the following statements? Lines of therapy are only relevant in the context of a palliative treatment, but not in the context of a curative therapy. |
| 1. Maintenance therapy consists a reduced drug regimen, but may also include new drugs. | Maintenance therapy: Maintenance therapy usually consists of a reduced drug regimen of the drugs previously administered, but may also include new drugs. |
| 1. Maintenance therapy initiated following systemic therapy is added into the existing LOT. | Maintenance therapy: If maintenance therapy, which typically involves a reduced drug regimen, is initiated following systemic therapy, it is added into the existing line of therapy. |
| 1. Maintenance therapy with a new drug results in a change of LOT. | Maintenance therapy: If maintenance therapy involves a new drug that has not yet been used in the previous systemic therapy, this results in a change of the line of therapy. |
| 1. For simultaneous radiochemotherapy, both radiotherapy and chemotherapy are part of LOT. | To what extent do you agree with the following statement? In the case of simultaneous radiochemotherapy, both radiotherapy and chemotherapy are part of the line of therapy. |
| 1. To what extent does surgery represent a separate LOT? | To what extent do the following therapeutic modalities (possibly in connection with other therapy modalities) represent a separate line of therapy? Surgery |
| 1. To what extent does radiotherapy represent a separate LOT? | To what extent do the following therapeutic modalities (possibly in connection with other therapy modalities) represent a separate line of therapy? Radiotherapy |
| 1. To what extent do regional treatment procedures represent a separate LOT? | To what extent do the following therapeutic modalities (possibly in connection with other therapy modalities) represent a separate line of therapy? Regional treatment procedures (e.g. TACE) |
| 1. How do you assess the role of a 30-day therapy interruption between the same therapy for low-grade tumors in relation to LOTs? | Based on the following hypothetical case, how do you assess the role of therapy interruptions in relation to lines of therapy? 30-day therapy interruption between the same therapy for low-malignant tumors |
| 1. How do you assess the role of a 180-day therapy interruption between the same therapy for low-grade tumors in relation to LOTs? | Based on the following hypothetical case, how do you assess the role of therapy interruptions in relation to lines of therapy? 180-day therapy interruption between the same therapy for low-malignant tumors |
| 1. How do you assess the role of a 30-day therapy interruption between the same therapy for high-grade tumors in relation to LOTs? | Based on the following hypothetical case, how do you assess the role of therapy interruptions in relation to lines of therapy? 30-day therapy interruption between the same therapy for high-grade tumors |
| 1. How do you assess the role of a 180-day therapy interruption between the same therapy for high-grade tumors in relation to LOTs? | Based on the following hypothetical case, how do you assess the role of therapy interruptions in relation to lines of therapy? 180-day therapy interruption between the same therapy for high-grade tumors |
| 1. How do you assess the role of a therapy interruption between two different therapies in relation to LOTs? | Based on the following hypothetical case, how do you assess the role of therapy interruptions in relation to lines of therapy? Therapy interruption between two different therapies |
| 1. How do you assess the role of a therapy interruption during therapy due to tumor progression/recurrence in relation to LOTs? | Based on the following hypothetical case, how do you assess the role of therapy interruptions in relation to lines of therapy? Therapy interruption during therapy due to tumor progression/recurrence |
| 1. How do you assess the role of a therapy interruption between two different therapies due to tumor progression/recurrence in relation to LOTs? | Based on the following hypothetical case, how do you assess the role of therapy interruptions in relation to lines of therapy? Therapy interruption between two different therapies due to tumor progression/recurrence |
| 1. Does progression of the primary tumor usually initiate a change of LOT? | Which of the following events usually initiate a change of a line of therapy? Progression of the primary tumor |
| 1. Does metastasis of the primary tumor usually initiate a change of LOT? | Which of the following events usually initiate a change of a line of therapy? Metastasis of the primary tumor |
| 1. Does occurrence of a recurrence usually initiate a change of LOT? | Which of the following events usually initiate a change of a line of therapy? Occurrence of a recurrence |
| 1. Does occurrence of relevant side-effects usually initiate a change of LOT? | Which of the following events usually initiate a change of a line of therapy? Occurrence of relevant side-effects |
| 1. Does patient's preference usually initiate a change of LOT? | Which of the following events usually initiate a change of a line of therapy? Patient's preference |
| 1. Does adding one or more new drugs to an existing drug regimen lead to a change of LOT? | Which of the measures mentioned, if applicable, would lead to a change in a line of therapy as a consequence of the events you selected in the previous question? Adding one or more new drugs to an existing drug regimen |
| 1. Does discontinuation of one or more drugs from an existing drug regimen lead to a change of LOT? | Which of the measures mentioned, if applicable, would lead to a change in a line of therapy as a consequence of the events you selected in the previous question? Discontinuation of one or more drugs from an existing drug regimen |
| 1. Does discontinuation of all drugs administered so far lead to a change of LOT? | Which of the measures mentioned, if applicable, would lead to a change in a line of therapy as a consequence of the events you selected in the previous question? Discontinuation of all drugs administered so far |
| 1. Does replacement of a drug with another drug considered equivalent lead to a change of LOT? | Which of the measures mentioned, if applicable, would lead to a change in a line of therapy as a consequence of the events you selected in the previous question? Replacement of a drug with another drug considered equivalent (from the same class of drugs) |
| 1. Does replacing the currently administered drugs with other drugs lead to a change of LOT? | Which of the measures mentioned, if applicable, would lead to a change in a line of therapy as a consequence of the events you selected in the previous question? Replacing the currently administered drugs with other drugs |
| 1. Does changing in dose lead to a change of LOT? | Which of the measures mentioned, if applicable, would lead to a change in a line of therapy as a consequence of the events you selected in the previous question? Change in dose |
| 1. Does changing in administration interval lead to a change of LOT? | Which of the measures mentioned, if applicable, would lead to a change in a line of therapy as a consequence of the events you selected in the previous question? Change in administration interval |
| 1. Does changing in the route of administration lead to a change of LOT? | Which of the measures mentioned, if applicable, would lead to a change in a line of therapy as a consequence of the events you selected in the previous question? Change in the route of administration |
| 1. Does an interruption of treatment lead to a change of LOT? | Which of the measures mentioned, if applicable, would lead to a change in a line of therapy as a consequence of the events you selected in the previous question? Interruption of treatment |
| 1. How do you assess the role of a local therapy in the midst of systemic therapy related to LOTs? | Based on the following hypothetical case, how do you assess the role of local therapies related to lines of therapy? Local therapy in the midst of systemic therapy |
| 1. How do you assess the role of a local therapy after the end of systemic therapy related to LOTs? | Based on the following hypothetical case, how do you assess the role of local therapies related to lines of therapy? Local therapy after the end of systemic therapy |
| 1. How do you assess the role of surgery followed by chemotherapy (adjuvant) related to LOTs? | Based on the following hypothetical case, how do you assess the role of local therapies related to lines of therapy? Surgery followed by chemotherapy (adjuvant) |

Abbreviations: LOT, line of therapy; LOTs, lines of therapy.

**Table B.2.** Binary variables, coefficients and interpretation of values.

| **Category** | **Information** |
| --- | --- |
| **Variables** | Events and measures for change of LOT |
| **Analysis method / coefficient** | CP CIs, normalized entropy |
| **Interpretation of values / mathematical definition** | In this survey’s binary questions each respondent indicated whether a certain event or action would result in a change in LOT. For each question, the proportion of affirmative responses was calculated. High agreement corresponds to proportions close to $0$ or $1$, while low agreement corresponds to proportions close to $0.5$. The uncertainty about the underlying population proportion was quantified via CP CIs. For the sake of comparability, normalized entropy (further discussed below under categorical variables) was also calculated. |

Abbreviations: CIs, confidence intervals; CP, Clopper-Pearson; LOT, line of therapy.

**Table B.3.** Orderable variables, coefficients, excluded responses and interpretation of values.

| **Category** | **Information** |
| --- | --- |
| **Variables** | Definition, criteria and relevance of LOT, maintenance therapy, radiotherapy and chemotherapy belonging to LOT, therapy modalities surgery, radiotherapy and regional treatment procedures, therapy interruptions |
| **Analysis method / coefficient** | GMD |
| **Setting aside certain responses** | Twenty of the question variables, either naturally ordered or permitting ordering by setting aside certain responses, were defined as follows:   - Agreement (definition and criteria of LOT, relevance across disease stages and for both palliative and curative treatment intentions, maintenance therapy, radiotherapy and chemotherapy belonging to LOT): “do not agree at all” < “rather do not agree” < “undecided” < “rather agree” < “completely agree”; while setting aside “cannot judge”. - Frequency (therapy modalities surgery, radiotherapy and regional treatment procedures): “never” < “only in particular cases” < “usually” < “always”; while setting aside “unsure” and “cannot judge”. - Relevance of LOT in clinical practice and research: “hardly relevant” < “somewhat relevant” < “relevant” < “extremely relevant”; while setting aside “never engaged with the subject”. - Therapy interruptions: “LOT remains same“ < “unsure” < “LOT changes after the interruption”; while setting aside “cannot judge”. |
| **Interpretation of values / mathematical definition** | These ordinal structures are important for analysis, as some distinct responses are closer to each other than others. To account for this proximity in analyzing agreement, the dispersion metric GMD was chosen. It is defined as the average distance between all paired responses to the question. Thus, GMD requires a notion of distance. Therefore, the ordinal responses categories were mapped to equidistant points the unit interval $\left[ 0,1 \right]$, with the lowest mapped to $0$ and the highest to $1$. Uncertainty was quantified via percentile bootstrap CIs.  GMD of $0$ represents complete unanimity. Maximal GMD represents dissension, achieved by two equally sized groups espousing viewpoints at opposite ends of the spectrum. Maximum diversity of opinion—i.e. an underlying distribution of equal probabilities across the answer options—lies between unanimity and dissension, and depends on the number of answer possibilities. To permit comparison of GMD values for questions with different numbers of possible answers, a transformation was defined and referred as standardized GMD (see Equation C1 and C2 in Additional file C).  To respect the ordered structure of the responses, miscellaneous responses (e.g., “cannot judge”) were set aside to calculate GMD. Therefore, the calculated GMD is an estimate of the consensus within the subset of respondents with orderable answers. To reflect the miscellaneous responses in our analysis, number and proportion of these responses are reported. |

Abbreviations: CIs, confidence intervals; GMD, Gini mean difference; LOT, line of therapy.

**Table B.4.** Categorical variables, coefficients and interpretation of values.

| **Category** | **Information** |
| --- | --- |
| **Variables** | Role of local therapy: local therapy during and after systemic therapy, surgery followed by adjuvant chemotherapy |
| **Analysis method / coefficient** | Normalized entropy |
| **Interpretation of values / mathematical definition** | This is a metric with possible values ranging from $0$ to $1$, where $0$ corresponds to unanimity and $1$ represents maximal diversity of opinions, achieved only with an equal number of responses across each possible answer. It is well-suited for this analysis, as it enables comparison of agreement across questions with varying numbers of response options. CIs were derived from Sison and Glaz’ simultaneous CIs for multinomial proportions (see Equation C3 in Additional file C).  Normalized entropy was calculated by excluding the answer types “unsure” and “cannot judge” from the analysis. |

Abbreviation: CIs, confidence intervals.

**Table B.5.** Statistical tests for all three question types.

| Based on the respective CI a statistical test of the null hypothesis that the underlying distribution of expert opinions is maximally diverse (i.e., a uniform distribution response options) is necessary. That is,  $H_{0}:Prob\left( X=c_{i} \right)=1/N \text{for all possible answers }c_{i}, i=1,\ldots,N.$ | |
| --- | --- |
| **Test for binary questions** | Reject $H_{0}$ if $0.5$ lies in the CI for the proportion of affirmatives in the underlying population, otherwise fail to reject $H_{0}$. |
| **Test for orderable questions** | Let $\tau$ be the theoretical GMD under a uniform distribution across response options. Then reject $H_{0}$ if $\tau$ lies in the CI for the GMD of the underlying population, otherwise fail to reject $H_{0}$. |
| **Test for categorical questions** | Reject $H_{0}$ if $1$ lies in the CI for the normalized entropy of the underlying population, otherwise fail to reject $H_{0}$. |
| **Remark regarding FDR and BH correction** | The original *p*-values needed for the correction were derived from respective CI method (see Equation C4 in Additional file C). For categorical variables, the *p*-values attained when excluding “unsure” and “cannot judge” from the normalized entropy calculation were used. This is because of interest in heterogeneity of answers among those taking a clear stance.  Since the BH correction of the calculated p-values in multiple comparisons at the 5% level did not alter the raw test results, the findings are presented unadjusted, using 95% confidence intervals. Consistent with the BH correction, the false discovery rate (type 1 error rate) is maintained at no more than 5%. |

Abbreviations: BH, Benjamini-Hochberg; CI, confidence interval; FDR, False discovery rate; GMD, Gini mean difference.

**Additional file C**

The GMD of a vector is the average distance between its pairs of entries. For a probability distribution, the GMD is defined as the expected distance between two random values, each independently drawn from the distribution. The GMD of a vector of observations is used to estimate the GMD of the probability distribution from which the observations are drawn. To clarify, the following definition was used.

1. Let $\mathbf{x}=\left( x_{1},\ldots,x_{n} \right)$ be a vector in $\mathbb{R}^{n}$. Then the **GMD** (or **mean absolute difference**) of $\mathbf{x}$ is defined as

$$\Delta\left( \mathbf{x} \right):=\frac{1}{\binom{n}{2}}\sum_{1\leq i<j\leq n} \left| x_{i}-x_{j} \right|.$$

1. Let $F$ be a probability distribution. The **GMD** (or **mean absolute difference**) of distribution $F$ is defined as

$$\Delta\left( F \right):=\mathbb{E}\left| X-Y \right|,$$

- where $X$ and $Y$ are independent random variables with the same distribution $F$.

Lemma 1 and Lemma 2 are used below to justify the standardization transformation to make GMDs of questions with different numbers of possible ordered response more comparable.

**Lemma 1 (GMD for discrete uniform distribution)**. For integer $N\geq2$, the GMD of the uniform distribution on the $N$ equally spaced points $\{\frac{k}{N-1}:k=0,\ldots,N-1\}$ is $\tau_{N}:=\frac{1}{3}\cdot\frac{N+1}{N}$. In particular, for $N=3$, this GMD is $\tau_{3}=4/9$; and for $N=5$, this GMD is $\tau_{5}=2/5$.

**Proof**. Let $X$ and $Y$ be independent random variables with a uniform distribution on the points $\{\frac{k}{N-1}:k=0,\ldots,N-1\}$, i.e. $\mathbb{P}\left( X=k/\left( N-1 \right) \right)=1/N$ for $k=1,\ldots,N-1$.

$$\begin{matrix} \mathbb{E}\left| X-Y \right| & =\sum_{i=0}^{N-1} \sum_{j=0}^{N-1} |\frac{i}{N-1}-\frac{j}{N-1}\mathbb{|\cdot P}\left( X=\frac{i}{N-1},Y=\frac{j}{N-1} \right) \\ & =\sum_{i=0}^{N-1} \sum_{j=0}^{N-1} \frac{\left| i-j \right|}{N-1}\mathbb{\cdot P}\left( X=\frac{i}{N-1} \right)\mathbb{\cdot P}\left( Y=\frac{j}{N-1} \right) \\ & =\frac{1}{N-1}\cdot\frac{1}{N}\cdot\frac{1}{N}\sum_{i=0}^{N-1} \sum_{j=0}^{N-1} \left| i-j \right|=\frac{1}{\left( N-1 \right)N^{2}}\cdot2\sum_{0\leq i<j\leq N-1} \left| i-j \right| \\ & =\frac{1}{\left( N-1 \right)N^{2}}\cdot2\sum_{j=1}^{N-1} \sum_{i=0}^{j-1} \left( j-i \right) \\ & =\frac{1}{\left( N-1 \right)N^{2}}\cdot2\sum_{j=1}^{N-1} \left( j^{2}-\sum_{i=0}^{j-1} i \right)=\frac{1}{\left( N-1 \right)N^{2}}\cdot2\sum_{j=1}^{N-1} \left( j^{2}-\frac{j\left( j-1 \right)}{2} \right) \\ & =\frac{1}{\left( N-1 \right)N^{2}}\cdot2\sum_{j=1}^{N-1} \left( \frac{j^{2}+j}{2} \right)=\frac{1}{\left( N-1 \right)N^{2}}\sum_{j=1}^{N-1} \left( j^{2}+j \right), \end{matrix}$$

where Gauss’s summation formula was used in the third-to-last line. The well-known sum of the first $N$ squares formula states $\sum_{j=1}^{N} j^{2}=N\left( N+1 \right)\left( 2N+1 \right)/6$. Thus, $\sum_{j=1}^{N-1} j^{2}=\frac{1}{6}\left( N-1 \right)N\left( 2N-1 \right)$. Using this and Gauss’s summation then yields

$$\begin{matrix} \mathbb{E}\left| X-Y \right| & =\frac{1}{\left( N-1 \right)N^{2}}\left( \frac{1}{6}\left( N-1 \right)N\left( 2N-1 \right)+\frac{N\left( N-1 \right)}{2} \right) \\ & =\frac{1}{\left( N-1 \right)N^{2}}\left( \frac{N\left( N-1 \right)\left[ \left( 2N-1 \right)+3 \right]}{6} \right) \\ & =\frac{1}{\left( N-1 \right)N^{2}}\left( \frac{N\left( N-1 \right)\left[ 2N+2 \right]}{6} \right) \\ & =\frac{1}{3}\cdot\frac{N+1}{N}. \end{matrix}$$

$$◼$$

**Lemma 2 (Maximum GMD for distributions on** $\left[ 0,1 \right]$**)**. Let $F$ be a probability distribution that (almost surely) takes values in $\left[ 0,1 \right]$. Then the GMD of $F$ is no greater than $1/2$. That is, $\Delta\left( F \right)\leq1/2$. If $F$ is the distribution with $1/2$ probability of $0$ and $1/2$ probability of $1$ (i.e., Bernoulli with parameter $1/2$), then $\Delta\left( F \right)=1/2$.

*Proof*. A proof of the inequality $\Delta\left( F \right)\leq1/2$ can be found at (https://math.stackexchange.com/users/34662/pre-kidney), pre-kidney. Maximum mean absolute difference of two iid random variables. The equality $\Delta\left( \mathrm{Bernoulli}\left( 1/2 \right) \right)=1/2$ follows immediately from the definition of GMD. $◼$

Note that, in this case, $\mathrm{Bernoulli}\left( 1/2 \right)$ represents an underlying population with one half of expert opinions on each opposing side of a spectrum.

Based on Lemma 1 and Lemma 2, the value $\tau_{N}$—representing maximal diversity of opinion—serves as benchmark for agreement analysis: GMD estimates below $\tau_{N}$ suggest more agreement, while values above $\tau_{N}$ suggest more dissension. The benchmark value $\tau_{N}$, however, depends on the number of $N$: indeed, $1/3<\tau_{N}\leq2$ and $\tau_{N}=\frac{1}{3}\cdot\frac{N+1}{N}$ falls monotonically in $N$. Therefore, it does not make sense to plot the GMDs of questions with different numbers of potential responses next to each other. A monotonic transformation $T$ is proposed and used that transforms GMD values $\Delta\left( \mathbf{x} \right)$, depending on question’s number of response options $N\geq3$, as follows:

- If $0\leq\Delta\left( \mathbf{x} \right)\leq\tau_{N}$, then stretch $\Delta\left( \mathbf{x} \right)$ to the same proportion of the interval $\left[ 0,2/3 \right]$, namely $T\left( \Delta\left( \mathbf{x} \right),N \right)=\frac{\Delta\left( \mathbf{x} \right)}{\tau_{N}}\cdot\frac{2}{3}$.
- If $\tau_{N}<\Delta\left( \mathbf{x} \right)\leq1/2$, then stretch $\Delta\left( \mathbf{x} \right)$ to the same proportional point on the interval $\left[ 2/3,1 \right]$, namely $T\left( \Delta\left( \mathbf{x} \right),N \right)=\frac{2}{3}+\frac{\Delta\left( \mathbf{x} \right)-\tau_{N}}{1/2-\tau_{N}}\cdot\frac{1}{3}$.
- If $\Delta\left( \mathbf{x} \right)>1/2$, then $T\left( \Delta\left( \mathbf{x} \right),N \right)=1$.

**Equation C.1. Definition of GMD.**

Abbreviations: GMD, Gini mean difference; GMDs, Gini mean differences.

Let $\tilde{\mathbf{x}}=\left( \tilde{x}_{1},\ldots,\tilde{x}_{n} \right)$ be a vector of ordered categorical responses $x_{j}\in\{c_{j}:j=0,\ldots,N-1\}$ with the ordering $c_{0}\prec\ldots\prec c_{N-1}$, where $N\geq3$ is the number of ordered categories.

Map the vector of ordered categories $\tilde{\mathbf{x}}$ to a vector $\mathbf{x}=\left( x_{1},\ldots,x_{n} \right)$ whose elements lie in the evenly spaced values $\{\frac{k}{N-1}:k=0,\ldots,N-1\}$ by setting $x_{j}:=\sum_{k=0}^{N-1} \frac{k}{N-1}\mathbf{1}\{x_{k}=c_{k}\}$ for $j=1,\ldots,n$.

Calculate the GMD $\Delta\left( \mathbf{x} \right)$. And standardized this GMD as $T\left( \Delta\left( \mathbf{x} \right),N \right)\in\left[ 0,1 \right]$, where $T\left( \cdot,N \right):[0,\infty)\to\left[ 0,1 \right]$ is given by

$$T\left( z,N \right):=\left\{ \begin{matrix} \frac{2}{3}\cdot\frac{z}{\tau_{N}}, & 0\leq z\leq\tau_{N}, \\ \frac{2}{3}+\frac{1}{3}\cdot\frac{z-\tau_{N}}{1/2-\tau_{N}}, & \tau_{N}<z\leq1/2, \\ 1, & 1/2<z, \end{matrix} \right.$$

where $\tau_{N}=\frac{1}{3}\cdot\frac{N+1}{N}$ (ie, $\tau_{N}$ is the GMD of the uniform distribution on $\{\frac{k}{N-1}:k=0,\ldots,N-1\}$; see Lemma 1).

**Equation C.2. Definition for calculation method of standardized GMD.**

Abbreviation: GMD, Gini mean difference.

Below the definition of normalized entropy of a random variable as well as the definition of normalized entropy of a vector are shown. Note that the normalized entropy of a vector $\mathbf{x}$ is the plug-in estimate of the population’s true normalized entropy via the observed response proportions in the vector.

1. Given a discrete random variable $X$ with a probability mass function $\mathbb{P}\left( X=x_{i} \right)=p_{i}$ for $i=1,2,\ldots,N$, the **Shannon entropy** $H\left( X \right)$ is defined as:

$$H\left( X \right):=-\sum_{i=1}^{N} p_{i}\log_{2}\left( p_{i} \right)$$

- The maximum possible entropy for a discrete random variable with $N$ possible outcomes occurs when the distribution is uniform, i.e., $p_{i}=\frac{1}{N}$ for all $i$. The maximum entropy $H_{\text{max}}$ is

$$H_{\text{max}}=\log_{2}\left( N \right)$$

- **Normalized entropy** $H_{\text{norm}}\left( X \right)$ is the ratio of the entropy of the distribution to the maximum possible entropy for the given number of outcomes, i.e.

$$H_{\text{norm}}\left( X \right):=\frac{H\left( X \right)}{H_{\text{max}}}=\frac{-\sum_{i=1}^{N} p_{i}\log_{2}\left( p_{i} \right)}{\log_{2}\left( N \right)}$$

- This normalization ensures that the entropy value lies within the range $\left[ 0,1 \right]$, where $0$ indicates no uncertainty (i.e., a deterministic distribution) and $1$ indicates maximum uncertainty (i.e., a uniform distribution).

1. Let $\mathbf{x}=\left( x_{1},\ldots,x_{n} \right)$ be a vector of values, each drawn from a discrete distribution with potential outcomes $c_{1},\ldots,c_{N}$. Denote the observed outcome proportions $\hat{p}_{i}=\frac{1}{n}\sum_{j=1}^{n} \mathbf{1}\{x_{j}=c_{i}\}$ for each $i=1,\ldots,N$. **Normalized entropy** $H_{\text{norm}}\left( \mathbf{x} \right)$ is defined as

$$H_{\text{norm}}\left( \mathbf{x} \right):=\frac{-\sum_{i=1}^{N} \hat{p}_{i}\log_{2}\left( \hat{p}_{i} \right)}{\log_{2}\left( N \right)}.$$

To generate CIs for normalized entropy, bootstrap techniques are not used. This is because the point estimator for normalized entropy is $1$ exactly when all observed proportions $\hat{p}_{i}=1/N$. Therefore, even if the true normalized entropy is $1$ and a large data set is available, the point estimate will generally fall below $1$. (In fact, this point estimate will always be lower than $1$ whenever the sample size is not divisible by $N$.)

Instead, the following simple, but potentially overly conservative technique is selected to generate CIs.

*Procedure*

1. Calculate 95% simultaneous CIs $C\subseteq\left[ 0,1 \right]^{N}$ for the probabilities drawing each category, i.e. if $p=\left( p_{1},\ldots,p_{N} \right)\in\left[ 0,1 \right]^{N}$ is the true vector of population probabilities for each respective category, then $\mathbb{P}\left( p\in C \right)\geq0.95$. Sison and Glaz’ simultaneous CIs are used when $N\geq3$. For $N=2$, a simultaneous CI is derived for $\left( p_{1},p_{2} \right)=\left( p_{1},1-p_{1} \right)$ from the CP CI for $p_{1}$.
2. Find the values $L:=inf\{H_{\text{norm}}\left( q \right):q\in C,\sum_{j=1}^{N} q_{j}=1\}$ and $U:=sup\{H_{\text{norm}}\left( q \right):q\in C,\sum_{j=1}^{N} q_{j}=1\}$. Then $\left[ L,U \right]$ is a valid 95% CI for normalized entropy, since $0.95\mathbb{\leq P}\left( p\in C \right)\mathbb{=P}\left( p\in C,\sum_{j=1}^{N} p_{j}=1 \right)\mathbb{\leq P}\left( L\leq H_{\text{norm}}\left( p \right)\leq U \right)$. Fortunately, both optimization problems are tractable. The maximization problem can be solved by (disciplined) convex programming [1]. For the minimization problem, Bauer’s principle [2] can reduce the problem to a search over the extreme points of $C$. Since $C$ has the form $\{q\in\left[ 0,1 \right]^{N}:q_{i}\in\left[ \mathcal{l}_{i},u_{i} \right]\text{ for all }i,\sum_{j=1}^{N} q_{j}=1\}$ and is, thus, a polyhedron and only has finitely many extreme points.

Regarding the inequalities in Step 2 above, the CIs are valid, but potentially overly conservative.

**Equation C.3. Definition of normalized entropy and creation of CIs for normalized entropy.**

Abbreviations: CI, confidence interval; CIs, confidence intervals.

Consider a statistical test of the form $H_{0}:\vartheta=v$ vs. $H_{1}:\vartheta\neq c$, where $\vartheta$ is the parameter of interest—the intention is to reject the null that it is $v$. Assume a method is given for generating $\left( 1-\alpha\right)\cdot100$% CIs $C_{\alpha}$ for the parameter $\vartheta$ for any level $\alpha\in\left( 0,1 \right)$. Then the value

$$p:=inf\{\alpha\in\left( 0,1 \right):v\notin C_{\alpha}\} \left( 1 \right)$$

is a valid *p*-value if

$$1-\alpha\leq1-\alpha'\text{ implies }C_{\alpha}\subseteq C_{\alpha'}. \left( 2 \right)$$

To see why, recall that a $\left( 1-\alpha\right)\cdot100$% CI $C_{\alpha}$ induces a level $\alpha$ test for $H_{0}$ vs. $H_{1}$. If the implication Equation 2 holds, then the induced level $\alpha$ tests satisfy the monotonicity requirement in ((https://math.stackexchange.com/users/34305/madprob), madprob. Two definitions of p-value), which then shows that Equation 1 is a valid *p*-value (with respect to the definition in Casella & Berger [3]).

Note that even if Equation 2 does not always hold, it will hold in most circumstances. So, CIs can be used to generate rough $p$-value estimates. To generate *p*-values from CIs in this study, the largest $\alpha\in\left( 0,1 \right)$ such that $v\in C_{\alpha}$ are find, which yields slightly larger (i.e., more conservative) *p*-values than Equation 1 under slight violations of Equation 2.

**Equation C.4. P-value calculations.**

Abbreviations: CI, confidence interval; CIs, confidence intervals.

**Additional file D**

**Figures**

*Figure D.1 about here*

**Figure D.1**. Proportion of affirmative responses and 95% confidence interval for all binary questions.

Note: For events and measures for change of LOT, the category ”I cannot judge this for any of the answers mentioned” was also included with proportion of zero (proportion: 0.00 [0.00; 0.06]) for the event and with low proportion (proportion: 0.10 [0.04; 0.21]) for the measure (Additional file D Table D.1).

Abbreviations: admin., administration; equ., equivalent; LOT, line of therapy; prim., primary; rel., relevant; Replacem., replacement.

*Figure D.2 about here*

**Figure D.2.** Descriptive and consensus results (95%-CIs) regarding surgery **(a)**, radiotherapy **(b)**, regional treatment procedures **(c)** as a separate LOT and radiotherapy and chemotherapy as part of LOT **(d)**.

Note: A value for GMD of $0$ corresponds to unanimity, $2/3$ to maximal uncertainty (i.e., uniform distribution across answers), and $1$ to maximal dissension (i.e., equally sized groups opining at opposite ends of the spectrum). The standardization transformation serves to make GMD values comparable across questions with different numbers of possible answers. The response categories “I cannot judge” and “I am unsure” were excluded as indeterminate responses for calculating GMD. This leads to a little lower N for calculating the consensus metrics in comparison to the N for descriptive statistics. For the presentation in the table, a short version of the questions was used. An overview of the short versions with the assigned detailed formulation for all questions is given in Table B1 in Additional file B.

Abbreviations: CI, confidence interval; GMD, Gini mean difference; LOT, line of therapy.

**Tables**

**Table D.1.** Proportions of affirmative responses regarding events and measures for change of LOT (binary responses).

| Question | N | Yes prop 95%-CP-CI | Yes count |
| --- | --- | --- | --- |
| Which of the following events usually initiate a change of a line of therapy? | | | |
| Progression of the primary tumor | 60 | 0.97 [0.88, 1.00] | 58 |
| Metastasis of the primary tumor | 60 | 0.92 [0.82, 0.97] | 55 |
| Occurrence of a recurrence | 60 | 0.87 [0.75, 0.94] | 52 |
| Occurrence of relevant side-effects | 60 | 0.77 [0.64, 0.87] | 46 |
| Patient's preference | 60 | 0.48 [0.35, 0.62] | 29 |
| I cannot judge for any of the given answers. | 60 | 0.00 [0.00, 0.06] | 0 |
| Which of the mentioned measures lead to a change of a line of therapy? | | | |
| Adding one or more new drugs to an existing drug regimen | 54 | 0.61 [0.47, 0.74] | 33 |
| Discontinuation of one or more drugs from an existing drug regimen | 54 | 0.41 [0.28, 0.55] | 22 |
| Discontinuation of all drugs administered so far | 54 | 0.69 [0.54, 0.80] | 37 |
| Replacement of a drug with another drug considered equivalent (from the same class of drugs) | 54 | 0.17 [0.08, 0.29] | 9 |
| Replacing the currently administered drugs with other drugs | 54 | 0.87 [0.75, 0.95] | 47 |
| Change in dose | 54 | 0.17 [0.08, 0.29] | 9 |
| Change in administration interval | 54 | 0.15 [0.07, 0.27] | 8 |
| Change in the route of administration | 54 | 0.07 [0.02, 0.18] | 4 |
| Interruption of treatment | 54 | 0.22 [0.12, 0.36] | 12 |
| I cannot judge this for any of the answers mentioned. | 60 | 0.10 [0.04, 0.21] | 6 |

Note: Even the value $0.5$, representing maximal divergence of opinion, lies in the respective 95% confidence interval for three questions: Patient's preference, adding one or more new drugs to an existing drug regimen, discontinuation of one or more drugs from an existing drug regimen.

Abbreviations: CP-CI, Clopper-Pearson-confidence interval; prop, proportion.

**Table D.2.** Non-standardized GMD for definition, criteria and relevance of LOT, maintenance therapy, therapy modalities and interruptions.

| Question | Valid N | Non-std. GMD (95%-CI) |
| --- | --- | --- |
| A line of therapy represents a largely self-contained therapy concept, chosen primarily based on various patient and tumor characteristics. It can include different therapy modalities and specific treatment goals, such as tumor control or cancer cure. | 60 | 0.17 [0.12, 0.21] |
| There are currently clear and uniform criteria in place to determine the lines of therapy even in rare and complex situations in the course of the patient. | 60 | 0.31 [0.25, 0.36] |
| How relevant do you consider the definition of lines of therapy in everyday clinical practice? | 60 | 0.26 [0.20, 0.31] |
| How relevant do you think it is to define the lines of therapy in research, e.g. in the context of studies? | 60 | 0.24 [0.19, 0.28] |
| Lines of therapy are only relevant in inoperable, locally advanced or metastatic cancers, but not in early stages of the disease. | 60 | 0.37 [0.29, 0.43] |
| Lines of therapy are only relevant in the context of a palliative treatment, but not in the context of a curative therapy. | 60 | 0.33 [0.24, 0.39] |
| Maintenance therapy usually consists of a reduced drug regimen of the drugs previously administered, but may also include new drugs. | 52 | 0.30 [0.19, 0.38] |
| If maintenance therapy, which typically involves a reduced drug regimen, is initiated following systemic therapy, it is added into the existing line of therapy. | 51 | 0.29 [0.20, 0.36] |
| If maintenance therapy involves a new drug that has not yet been used in the previous systemic therapy, this results in a change of the line of therapy. | 51 | 0.37 [0.27, 0.43] |
| In the case of simultaneous radiochemotherapy, both radiotherapy and chemotherapy are part of the line of therapy. | 54 | 0.22 [0.10, 0.32] |
| To what extent do the following therapeutic modalities (possibly in connection with other therapy modalities) represent a separate line of therapy? | | |
| Surgery | 51 | 0.33 [0.26, 0.38] |
| Radiotherapy | 50 | 0.28 [0.21, 0.34] |
| Regional treatment procedures (e.g. TACE) | 49 | 0.31 [0.24, 0.37] |
| Based on the following hypothetical case, how do you assess the role of therapy interruptions in relation to lines of therapy? | | |
| 30-day therapy interruption between the same therapy for low-grade tumors | 53 | 0.07 [0.00, 0.16] |
| 180-day therapy interruption between the same therapy for low-grade tumors | 51 | 0.43 [0.33, 0.49] |
| 30-day therapy interruption between the same therapy for high-grade tumors | 52 | 0.21 [0.08, 0.32] |
| 180-day therapy interruption between the same therapy for high-grade tumors | 49 | 0.47 [0.40, 0.50] |
| Therapy interruption between two different therapies | 49 | 0.06 [0.00, 0.13] |
| Therapy interruption during therapy due to tumor progression/recurrence | 51 | 0.50 [0.44, 0.51] |
| Therapy interruption between two different therapies due to tumor progression/recurrence | 52 | 0.09 [0.00, 0.19] |

Note: GMD of $0$ represents complete unanimity. A maximal GMD represents dissension, achieved via two equally sized groups espousing viewpoints at opposite ends of the spectrum. Non-standardized GMD is calculated for questions with orderable responses.

Abbreviations: GMD, Gini mean difference; Non-std., non-standardized.

**References Additional files**

1. Fu A, Narasimhan B, Boyd S. CVXR: An R Package for Disciplined Convex Optimization. *J. Stat. Softw*. 2020;94. https://doi.org/10.18637/jss.v094.i14

2. Bauer H. Minimalstellen von Funktionen und Extremalpunkte. *Arch.* Math*.* 1958;9:389-93. https://doi.org/10.1007/BF01898615

3. Casella G, Berger R. *Statistical inference*. 2th ed. Boca Raton, Florida: CRC Press; 2002. https://doi.org/10.1201/9781003456285
